# Transition Radiation Field Enhanced Laser Proton Acceleration Employing Near-Critical-Density Foam

**DOI:** 10.1038/s41467-026-74298-0

**Published:** 2026-06-15

**Authors:** C. Y. Qin, X. S. Geng, H. Zhang, L. H. Yu, L. G. Zhang, Y. Z. Dai, J. Wang, B. W. Zhang, X. J. Guo, D. R. Xu, S. Xu, C. L. Ding, Y. Xu, Y. Q. Liu, C. Wang, B. N. Shi, Z. X. Zhang, X. Y. Liu, Y. X. Leng, X. Y. Liang, B. F. Shen, L. L. Ji, R. X. Li

**Affiliations:** 1 State Key Laboratory of Ultra-intense Laser Science and Technology, Shanghai Institute of Optics and Fine Mechanics, Chinese Academy of Sciences, Shanghai, 201800 China; 2 CAS Center for Excellence in Ultra-intense Laser Science, Shanghai, 201800 China; 3 Center of Materials Science and Optoelectronics Engineering, University of Chinese Academy of Sciences, Beijing, 100049 China; 4 Zhangjiang Laboratory, Shanghai, 201210 China; 5 Department of Physics, Shanghai Normal University, Shanghai, 200234 China; 6 ShanghaiTech University, Shanghai, 201210 China

**Keywords:** Plasma-based accelerators, Terahertz optics, Laser-produced plasmas

## Abstract

Laser-driven protons with ultrafast temporal properties attract great interest in fields ranging from flash radiation oncology to compact accelerators. High-efficiency energy coupling of protons from laser-induced accelerating fields is complex, hybrid acceleration mechanisms that combine multiple field contributions prove critical for optimizing proton energy. Here, we report a laser proton acceleration scheme in which proton energy can be enhanced by a transition radiation field (TRF) built by high-energy and large-charged electron bunches. Using near-critical-density plasmas, we experimentally produce electron beams with charges up to ~30 nC (>13 MeV). As these electrons exit the target, they emit intense TRF with energy up to 0.6 J in 0.1-15 THz range, corresponding to an acceleration field of 10^12–13^ V m^–1^. When superposed with the charge-separation field (CSF), proton cut-off energy is boosted by more than a factor of two, reaching 90 MeV. The resulting spectra exhibit a distinctive plateau-shaped feature in the high-energy regime. Multi-dimensional kinetic simulations confirm the synergistic role of the TRF and CSF in both enhancing the proton energy and shaping the spectral structure. This scheme provides new insights into the coupling between relativistic electron beams and acceleration fields and facilitates more efficient laser-driven proton acceleration.

## Abstract (web summary)

Recent developments of proton acceleration have demonstrated remarkable energies, yet further insight into the acceleration mechanism is needed to fully achieve application-relevant regimes. Here, the authors demonstrate a hybrid scheme where the established target normal sheath acceleration mechanism is enhanced by transition radiation fields.

## Introduction

Laser-driven proton sources have been extensively studied because of their potential applications in probing high-energy-density states^1^, radiation oncology^2^, fast ignition fusion^3^, and laser nuclear physics^4^. Understanding and enhancing proton acceleration to achieve higher energies is essential for advancing these applications. Over the past two decades, steady progress has been made by optimizing laser parameters and tailoring target designs. This has resulted in proton energies approaching 100 MeV, primarily through the target normal sheath acceleration (TNSA) mechanism or radiation pressure acceleration (RPA) combined with TNSA^5–7^. Recently, the rapid development of femtosecond petawatt-class lasers, which typically offer higher laser intensities and repetition rates^8^, has shown great potential in proton acceleration. Proton beams with energies ranging from 60 to 93 MeV can be consistently produced^9–12^. At laser intensities of 10^21–22^ W cm^–2^, proton beams with energy > 100 MeV are produced via hybrid acceleration mechanisms, such as joint acceleration of RPA and Coulomb repulsion in finite size targets^13^, and cascaded acceleration accompanying relativistic-induced transparency using ultrathin targets^14^.

Improving laser-driven proton acceleration also relies on a thorough understanding of the coupling mechanisms between the laser field, energetic electrons, and the accelerating field. In the TNSA mechanism, protons gain energy in a quasi-static charge separation field (CSF), which is a localized sheath electric field established by ponderomotive electrons^15–17^. The acceleration can be greatly improved by employing specially designed targets, such as near-critical-density (NCD) targets^18–24^ and micro-structured targets^25,26^, which enhance laser energy deposition and facilitate the generation of large-charged superponderomotive electron (SPE) beams via direct laser acceleration (DLA)^27–29^. These SPEs are shown to increase the proton acceleration energies and efficiencies by strengthening the sheath field at target rear^18–23^. Notably, carbon nanotube foam (CNF), a sparse three-dimensional solid composed of nanotubes with diameters of several tens of nanometers, exhibits densities 2–3 orders of magnitude lower than solid materials. This unique architecture allows for the creation of homogeneous NCD plasma layers with tunable thicknesses ranging from several microns to hundreds of microns^30^. When irradiated by intense laser pulses, CNF targets enable the generation of high-charge relativistic electron beams. Experiments have shown that CNF can achieve a ~ 2 times increase in proton energy and enhance heavy-ion acceleration through improving the strength of the acceleration field or the acceleration time^18,19,31^. However, it is still unclear how the energy of SPEs is coupled into the sheath field.

In this work, we present a hybrid proton acceleration scheme in which the TNSA proton energy is enhanced via the transition radiation fields (TRF) generated by high-energy DLA electron beams produced in CNF targets. When the DLA electron beam transverses the rear surface of the substrate target, strong TRF is emitted with longitudinal electric field approximated by ${E}_{x}\approx \,{{{\rm{\hbox{-}}}}}Q/(4{{{\rm{\pi }}}}{\varepsilon }_{0}R)$^32^. Here $Q$ is the linear charge density (charge per unit length), $R={ct}$ is the radius of light cone and ${\varepsilon }_{0}$ is the vacuum permittivity. We notice that this electric field becomes comparable to the CSF with sufficiently large beam charge. For a 5 nC electron bunch with 30 fs duration, the TRF near the target rear reaches 10^12 ^V m^–1^. This field will exert non-negligible acceleration effects on protons. In our experiments, electron beams with charge up to 26 nC (kinetic energy >13 MeV) are produced. The resulting TRF is measured in the terahertz regime to carry sub-joule energies, corresponding to acceleration fields in the range of 10^12–13^ V m^–1^. This TRF jointly boosts the proton cut-off energy to 90 MeV, more than twice the energy obtained using TNSA alone. The proton spectra exhibit a long plateau structure extending to the high-energy end, markedly distinct from the Maxwellian profile typical of TNSA. Based on three-dimensional relativistic kinetic simulations, we decouple the TRF from the CSF, and that suggests TRF plays a non-negligible role in both enhancing the peak field strength and broadening the acceleration region. The onset of TRF offers a promising strategy for optimizing laser-proton acceleration across various applications.

## Results

### Experimental results

**Fig. 1:**
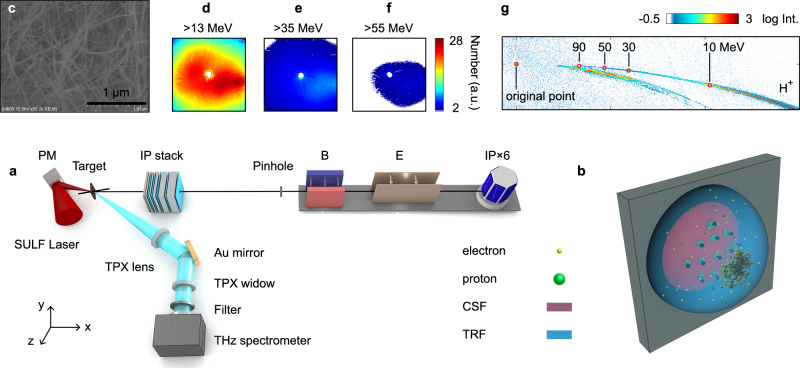
Sketch of the experimental setup and example measurements. **a** The laser pulse is reflected by a PM and focused onto the target surface with a 10^°^ incidence angle. The particle diagnostic devices are arranged along a direction of 5^°^ off the normal line behind the target. A multichannel spectrometer is used to measure the THz emission collected by a lens at an angle of 20° off the laser direction. It should be noted that due to space constraints, the IP stack and THz spectrometer were not used simultaneously. **b** Mechanism drawing of the transition radiation field enhanced proton acceleration. **c** SEM image of the carbon nanotube foam. **d–f** Typical electron profile distribution of the 35 μm CNF target obtained from the IP stack. **g** Raw IP data measured by the TPS.

The experiments were performed at the Shanghai Superintense Ultrafast Laser Facility (SULF)^33^. The *p*-polarized 30-fs (full width at half maximum, FWHM) laser pulse from a Ti: sapphire laser system (central wavelength *λ* = 800 nm) was focused by an *f* / 2 off-axis parabolic mirror to a spot of 4.3 μm (1/*e*^2^ diameter). As shown in Fig. 1a, after reflection by a plasma mirror (PM), the laser pulse was directed onto the target surface at 10^°^ incident angle, resulting in an on-target intensity of ~5 × 10^21 ^W cm^–2^ with 56 J energy. To measure the charge, spectrum, and profile of the electron beam, a stack of image plates was positioned 5 cm behind the target, oriented along the 5^°^ direction between the normal and laser directions. Following that, a Thomson parabola spectrometer (TPS) was set at 90 cm. A multichannel THz spectrometer^34^ located at target side direction aiming to measure the THz spectrum in independent shots.

Low-density CNFs with thickness ranging from 20 to 120 μm are fabricated on 100 nm thick SiN film using chemical vapor deposition method^30,35^. Figure 1c shows the SEM image of CNF, the CNF bulk density is around 7 mg cm^–3^, corresponding to an electron density of ~1 *n*_*c*_ if the carbon atoms are fully ionized, where *n*_c_ *= m*_e_*ε*_0_*ω*^2^/*e*^2^ is the critical density of plasma (*m*_e_, *e* and *ω* are the electron mass, electron charge, and laser frequency, respectively). Density is characterized by measuring the mass per unit volume as described previously^30^. Since our laser intensity is much higher than the relativistic threshold *a*_0_ = 1 (*a*_0_ is the normalized amplitude of the laser), the laser pulse can travel a long distance in the CNF without being reflected.

**Fig. 2:**
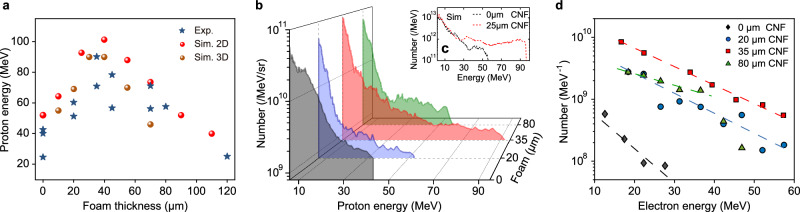
Proton cutoff energy and energy spectra of protons and electrons. **a** Maximum energy of protons obtained from targets with different CNF thicknesses. The blue stars, red, and orange dots represent the experimental, 2D, and 3D simulation results for proton energy, respectively. **b** Typical proton energy spectra of SiN substrate and CNF targets. **c** Energy spectra of protons from simulations. **d** Electron energy spectra corresponding to the cases in (**b**).

Figure 2a shows the proton cutoff energy according to the TPS data for varying CNF thicknesses. As thickness increases, proton energies increase steeply, then decrease gradually. Under our experimental conditions, optimal acceleration is achieved at CNF thickness of approximately 35 μm, where the maximum proton energy reaches 90 MeV (the raw IP image is shown in Fig. 1g). The average energy at this thickness is 80.3 MeV, which is about 2.3 times as that of a flat target (35.8 MeV). For thinner or thicker foams, the increase in proton energy is less significant. When CNF exceeds 100 μm, the acceleration enhancement diminishes, resulting in proton energies even lower than those obtained from the flat substrate.

**Fig. 3:**
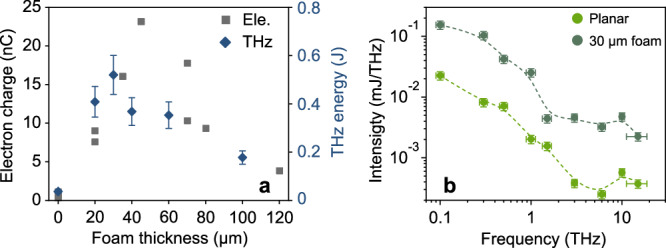
Measurements of electron and THz pulse. **a** Electron charge (>16.2 MeV) and THz energy (0.1–15 THz) under varying foam target thickness. The total THz energy is obtained by integrating over the entire spectrum. **b** Typical THz frequency spectra of planar and foam targets measured by the multichannel spectrometer. The terahertz signals were diagnosed independently in separate shots with laser energy of 68 J. The uncertainty in THz pulse energy measurements was mainly determined by the peak and average transmittance of the bandpass filters.

The proton energy enhancement can be traced to the electron behavior. Figures 2d and 3a show the electron energy spectra and charge, respectively. For the 35 μm CNF target, the charge is estimated to be as large as 26 nC for electrons with energy > 13 MeV. The slope temperature is about 15 MeV according to the exponential fitting, which is much higher than that of 100 nm SiN (7.9 MeV). This is consistent with the trend of the proton energy enhancement. Figures 1d–f show the typical electron distribution, where more electrons tend to be distributed along the laser direction, consistent with the characteristics of DLA.

Aside from the proton energy enhancement, we observe a unique feature in the proton spectra of CNF targets, as shown in Fig. 2b. The typical Maxwellian distribution is followed by a long plateau towards the high-energy end. For the 35 μm CNF target, the plateau region covers 60–90 MeV with a total span of 30 MeV. This is drastically different from the spectrum in the TNSA acceleration, which usually exhibits an exponentially decaying distribution. We find that it is not a feature inferred solely from the enhancement of the TNSA field, since the latter is believed to increase the proton population and cutoff energy rather than altering the spectral shape. Similar plateau spectra have been observed in magnetic vortex acceleration (MVA)^36,37^. Typical MVA requires the large-scale low-density plasma behind the target^36^. Here, the 500 *n*_c_ substrate blocks the laser penetration and attenuates the magnetic field expanding from the plasma channel to the proton acceleration region. Moreover, MVA protons are highly collimated, with a typical full angle of 1–3° for high-energy protons when using a high-power laser^38,39^. In our experiment, the TPS was configured to diagnose only protons emitted at an angle of 5° with respect to the laser propagation direction.

It is well-known that energetic electrons transmitted from the plasma boundary to the vacuum are accompanied by efficient TRF. This radiation is usually in the THz regime when using femtosecond-laser-drivers^40,41^. In the case of monoenergetic electron bunch, the total coherent TRF energy over all angles and frequencies is given by W_tot_ = (4*r*_e_*m*_e_*c*^2^)*N*^2^*ln*(*γ*)^42^, where *r*_e_, *N* and *γ* are classical electron radius, electron number and relativistic factor, respectively. One thus envisages that with sufficiently large population of high-energy electrons the TRF could be strong enough to contribute to proton acceleration. In the separate shots with higher laser energy of 68 J, we measured the TRF spectrum in frequency band of 0.1-15 THz as shown in Fig. 3. We find that the trend of radiation energy with respect to the foam thickness shows good correlation to the electron charge, which is also consistent with that of proton cutoff energy in Fig. 2a. Apparently, the THz intensity of CNF target is one order of magnitude higher than planar target.

For the foam target with a thickness of 30 μm, the generated THz radiation energy is 519.8 ± 80.8 mJ, corresponding to an energy efficiency of 0.76 ± 0.12%. Assuming the same conversion efficiency holds, the THz energy under the 56 J laser condition is estimated to be 425.6 ± 67.2mJ. The measured spectrum shows a central frequency corresponding to a wavelength of approximately 27 μm (see the Methods for details). Following the estimation of ref. ^43^, we estimate the THz pulse duration to be approximately *τ*_THZ_ ~ 83 ± 22 fs, corresponding to 2*τ*_L_ – 3.5*τ*_L_ (*τ*_L_ is the laser pulse duration). The contribution of the TRF to the accelerating field can be estimated as ${{{\rm{TRF}}}}\approx \sqrt{\frac{2{W}_{{THz}}}{{\varepsilon }_{0}c{\pi \tau }_{{THz}}{r}_{{THz}}^{2}}}$, where W_THz_ and *r*_THz_ are the energy and transverse radius of TRF, respectively. The acceleration field is highly sensitive to the source size (${{{\rm{TRF}}}}\propto \frac{1}{{r}_{{THz}}}$). At the target rear surface, the source size is comparable to the diameter of the plasma channel within the foam. Simulations indicate a transverse size of *r*_THz_ = 5 μm. Here, considering measurement uncertainties in both energy and duration, the resulting accelerating field is estimated as *E*_acc_ = (7.0 ± 2.2) × 10^12 ^V/m. As a result, protons can gain effective acceleration at 4.8–9.2 MeV μm^–1^ by the TRF in the initial stage.

Accurate estimation of TRF strength relies on more precise measurements. Here, while exact field strengths may have uncertainty, the qualitative scaling with electron charge and foam thickness is robust. It is noteworthy that previously observed THz intensities (typically in GV m^–1^ regimes) have primarily been employed to accelerate lighter particles like electrons^44–47^ and positrons^48,49^, our results demonstrate the feasibility of scaling the TRF strength by three orders of magnitude to accelerate protons, despite their mass is ~2000 times greater than electrons.

### Theoretical calculation of TRF

To interpret the experimental observations at the fundamental level, we perform rigorous theoretical calculations of the TRF. When a strong laser impinges onto a plasma foil, electrons near the front surface are heated and the hot electrons expand and traverse the foil to the back surface. The process can be regarded as the diffusion of an electron-ion plasma in thermal equilibrium^50^. The hot electrons are much faster than the ions, therefore creating CSF near the rear surface to accelerate the ions. Some of these electrons get reflected by the CSF, meanwhile, the high-energy electrons escape the target rear, mainly contributing to transition radiation (such as shown in Figures S2–S4 of the Supplementary file). Therefore, during the laser-foil interaction, both the CSF and TRF coexist. In the laser-foil case, however, since the number of high-energy electrons is relatively small, the TRF is generally weak, and its contribution to proton acceleration can be neglected.

The traversal of an electron across the plasma boundary can trigger TRF due to the change in dielectric constant. While the Coulomb field moves with the electron, the TRF is radiated and decoupled from the electron. When the electron is relativistic, the Coulomb field and TRF overlap with each other. It is difficult to directly decouple the two fields for relativistic electrons in particle-in-cell (PIC) simulations. Therefore, a post-processing method is required to analytically decompose the corresponding longitudinal Coulomb field and TRF based on the electron dynamics obtained from PIC simulations. By superimposing the theoretical fields of individual particles, the collective CSF and radiation field are revealed. The advantage of this approach is that it decouples the two field components even in the relativistic regime. In the weakly relativistic case, the CSF obtained via this method should be consistent with that derived from Poisson’s equation.

Following the work by Carron^32^, one is able to calculate and decompose the fields into Coulomb part and radiation part for an electron bunch exiting a conducting plane in the relativistic regime. In our case, the plasma can be regarded as good conductors, and the solution of the longitudinal electric field is

1 $${E}_{x}=\frac{Q}{{\gamma }^{2}}\left(\frac{1}{{S}_{-}}+\frac{1}{{S}_{+}}\right)-\frac{2Q}{R},0 < T < {\tau }_{{beam}}$$

2 $${E}_{x}=\frac{Q}{{\gamma }^{2}}\left(\frac{1}{{S}_{-}}-\frac{1}{{S}_{{l}_{-}}}+\frac{1}{{S}_{+}}-\frac{1}{{S}_{{l}_{+}}}\right),T > {\tau }_{{beam}}$$

The Eqs. (1) and (2) correspond to retarded time within the pulse length of the beam and outside the pulse length of the beam. Here, $Q$ is the bunch charge divided by bunch length, ${S}_{\pm }=\sqrt{{\left({vt}\pm x\right)}^{2}+\frac{{r}^{2}}{{\gamma }^{2}}}$, ${S}_{{l}_{\pm }}=\sqrt{{\left({vt}-l\pm x\right)}^{2}+\frac{{r}^{2}}{{\gamma }^{2}}}$, $R={ct}$ is the light cone, $T={\tau }_{{beam}}-R/c$ is the retarded time, $v$ is the velocity of the beam, ${\tau }_{{beam}}$ is the beam duration, $l=v{\tau }_{{beam}}$ is the beam length and $r=\sqrt{{y}^{2}+{z}^{2}}$ is the radius from the *x*-axis, respectively. The parameters ${S}_{\pm }$ account for the retarded positions of the beam and its image, capturing the finite propagation time of electromagnetic disturbances. From the above equations, one can immediately find that the dominant term $-2Q/R$ in Eq. (1) represents the radiation field that decays as $1/R$ and extends far beyond the ordinary sheath scale, and the terms with $1/{\gamma }^{2}$ prefactors describe the Coulomb-like space-charge fields of the bunch, which are strongly suppressed for relativistic electrons. In our modeling, we treat each macro particle in the simulation as a small bunch with a length of the cell size. By summing up all the $-2Q/R$ term and $1/{\gamma }^{2}$ terms of each macro particle, we will get the collective radiation field and longitudinal Coulomb field of an electron bunch.

### Simulation of TNSA-TRFA hybrid acceleration

**Fig. 4:**
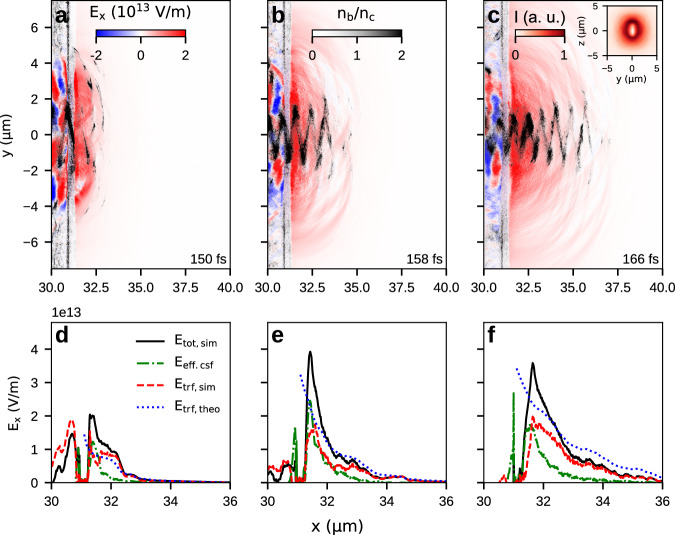
Evolution of the electron beam and corresponding acceleration field. **a–c** Evolution of the acceleration field *E*_x_ and electron beam transitioning through the foil. The insert plots the average intensity distribution that incorporates the full three-dimensional information of the electric field. **d–f** On-axis *E*_x_. Total field from simulation (*E*_tot,sim_, black-solid line), the effective CSF *E*_eff.csf_ (green-dash-dotted line), their difference *E*_trf,sim_ = *E*_tot,sim_ - *E*_eff.csf_ (red-dashed line), and the TRF *E*_trf,theo_ calculated from Eqs. (1–2) (gray-dotted line).

**Fig. 5:**
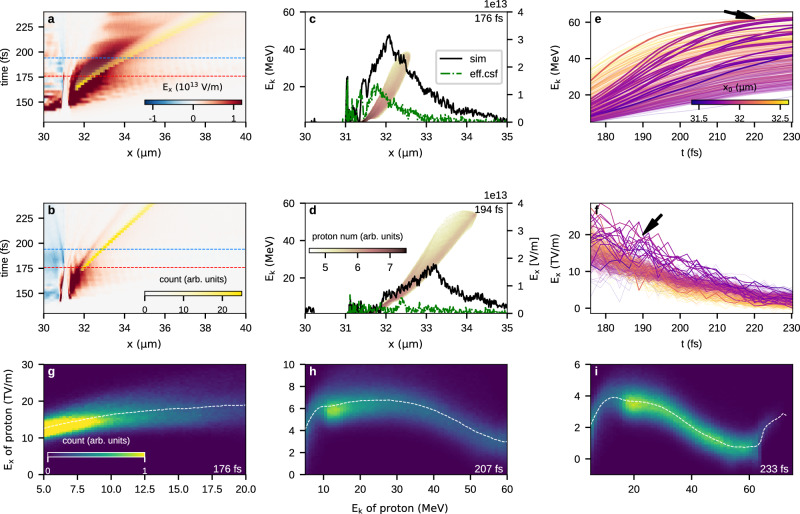
Time evolution of acceleration field intensity and protons distribution. **a, b** Evolution of the near-axis *E*_x_ field (blue-red) and the proton (>10 MeV) distribution (white-yellow) for **a** the TRF c**a**se (*E*_tot,sim_) and **b** the effective CSF case (*E*_eff.csf_). **c, d** Sliced near-axis *E*_x_ fields of **a**, **b** and proton distribution in *x*-*E*_k_ space at 176 fs (red lines) and 194 fs (blue lines), corresponding to the blue-dashed and red-dashed lines in (**a, b**). **e, f** The evolution of the energy and the witness *E*_x_ of protons. The color corresponds to the initial *x* of protons at 176 fs. **g–i** Proton distribution in the *E*_k_–*E*_x_ space at 176 fs, 207 fs, and 233 fs. The white-dashed lines are the average *E*_x_ fields at given *E*_k_.

We perform three-dimensional PIC simulations with parameters similar to experiments to find out the role of TRF in enhanced proton acceleration. We trace the DLA electrons from the NCD plasma and derive the real-time TRF following the above method. The total acceleration field *E*_x_ and the electron bunch from DLA are shown in Fig. 4a–c for 30 μm foam thickness. The periodic structure of the electron bunch is a typical sign of DLA, with a spacing of half of the laser wavelength (0.4 μm) on each side. When they exit the plasma target, a strong radiation field is formed and co-moves with the electron bunch. The field peaks near the rear of the target, i.e., at about 31.5 μm, according to Eq. (1) and diverges spherically as it further propagates. It thus extends to a region much larger than that of the known CSF, especially in Fig. 4b and c. Also, the hollow structure of the field intensity in Fig. 4c aligns with the characteristics of TRF^42,45,51^. As the TRF overlaps with the CSF, a simulation including only laser-substrate interaction (without foam) is considered to extract the effective CSF *E*_eff.csf_ (see the Methods for details). The contribution of TRF is thus revealed by subtracting the *E*_eff.csf_ from the total acceleration field *E*_tot,sim_. In Figs. 4d–f, we compare the on-axis distributions of *E*_tot,sim_, *E*_eff.csf_ and the approximated TRF *E*_trf,sim_ = *E*_tot,sim_ - *E*_eff.csf_, together with the TRF *E*_trf,theo_ derived via Eqs. (1) & (2). We see that the derived TRF agrees well with the TRF approximated from simulation in Fig. 4d when the bunch begins exiting the plasma rear. As the bunch propagates further, the shape and magnitude of both are still consistent in Fig. 4e, f. The slight difference originates from the assumption that the electrons propagate with uniform speed when calculating TRF while it expands as it propagates. The amplitude of TRF is comparable to the CSF all along, reaching 10^13 ^V m^–1^. A most significant role of TRF is the expansion of the acceleration region as shown by the widths in Fig. 4f. The range of the CSF stay at about 0.5 μm at FWHM, nearly unchanged during the whole acceleration process (also shown in Fig. 5b), which is basically CSF restricted near the target. When compared to the full simulation, one can see that the acceleration range expands to more than 1 μm at FWHM (black-solid line in Fig. 4f) due to the existence of the TRF, since it is a radiation field capable of propagating for longer distance than CSF. Therefore, the TRF not only doubles the acceleration gradient by directly exerting on the TNSA protons, but also extends the acceleration range, the acceleration time and the number of accelerated protons. A schematic illustrating this acceleration process is provided in Fig. 1b.

Next, we investigate the formation of the plateau region of the proton spectrum. In general, TNSA protons have a Maxwellian spectral profile. This is because in the well-known plasma expansion model of TNSA^50^, the proton front, where the most energetic protons sit, moves with the peak of the sheath field. Behind the peak is the declining region of the sheath field, where protons of lesser energies are located. As a result, protons of higher energies always witness stronger sheath field, forming a self-similar evolution and hence Maxwellian spectral profile. In our case, the acceleration range and duration of TRF are much longer than CSF, since the duration and length of TRF is determined by the DLA electron bunch which inherits the laser pulse duration, as shown by the evolution of the near-axis ${E}_{x}$ field and proton (>10 MeV) distribution are compared to the CSF case in Fig. 5a, b. It is clear that both the scale and the duration of *E*_tot,sim_ is much larger than *E*_eff.csf_. It offers an acceleration region that is significantly larger than in the pure CSF case.

The extended acceleration range and duration enable sustained acceleration of low-energy protons. We take the *x*-*E*_k_ distribution at 176 fs and 196 fs and display them in Fig. 5c, d. At 176 fs, when the acceleration begins, protons in both the TRF and CSF cases experience the peak of the acceleration field near 32 μm. As the substrate and the CSF expand, the high-energy protons no longer overlap with the strongest part of CSF since these protons expand faster than the substrate. Therefore, at 196 fs, the acceleration gradient drops significantly in the CSF case as shown in Fig. 5b. Nevertheless, in the TRF case, the mid-low protons still experience a strong acceleration field near *x* = 33 μm due to the extended acceleration range through TRF. Eventually, these protons catch up with the ones of higher energies. In other words, the number of protons in the high energy region grows, leading to a flattened spectrum profile in Fig. 2b. This process is rendered in the evolution of *E*_k_ and acceleration gradient in Fig. 5e, f where trailing mid-low energy protons experience high acceleration gradient (arrow in Fig. 5e) and get accelerated to mid-high energies (arrow in Fig. 5f).

This dynamic process is further elucidated by examining the acceleration gradient experienced by protons of different energies, as shown in the *E*_k_-*E*_x_ phase-space distributions in Fig. 5g–i. At 176 fs, when acceleration begins, higher-energy protons experience stronger longitudinal electric fields, resulting in a Maxwellian-like energy spectrum (Fig. 5g), This is consistent with typical TNSA behavior. By 207 fs, the acceleration gradient for the high-energy protons diminishes more rapidly than for the lower-energy ones. This trend reflects the influence of the TRF, which effectively extends both the spatial and temporal reach of the acceleration zone, as also seen in Fig. 5d. As a result, mid-low-energy protons continue to be accelerated over a longer period, gradually filling the high-energy tail of the spectrum. By 233 fs, the phase-space distribution becomes nearly uniform over the 20–60 MeV range (Fig. 5i), indicating the establishment of a broad plateau in the energy spectrum, which is a hallmark feature of TRF-assisted acceleration.

## Discussion

The simulation results for the proton cutoff energy are summarized in Fig. 2a and show reasonable agreement with the experimental measurements. Due to the prohibitively high computational cost of modeling thick foam targets in 3D, cutoff energies for CNF thicknesses greater than 70 μm are determined from reference 2D simulations.

**Fig. 6:**
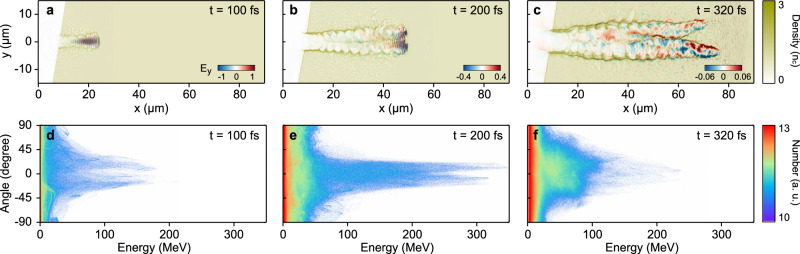
Temporal evolution of the laser and electron beam. **a–c** Distribution of the laser field and electron density in CNF at different times. They are normalized to the laser electric field E_0_ and critical density *n*_c_, respectively. **d–f** Angular distribution of electron beams.

An optimal CNF thickness facilitates the formation of a stable plasma channel that guides the laser pulse effectively. As shown in Fig. 6, the laser begins to bifurcate after approximately 40 μm of propagation, aligning well with the experimentally identified optimal target thickness. At this moment, the electron beam exhibits optimal collimation and energy, while at earlier (t = 100 fs) or later (t = 320 fs) temporal points, both parameters deteriorate obviously. When the CNF target is too thin, the laser-foam interaction volume is limited, leading to insufficient energy absorption and transfer; the energy and number of electron beams are constrained. If the CNF is excessively thick, the laser undergoes filamentation or bifurcation during propagation and is eventually absorbed within the foam. Although the accelerated electron beam can still propagate forward, it experiences strong divergence. These quality degenerates of the electron beam, in turn, will consequently degrade the near-field intensity of the transition radiation. Accordingly, the balance between sufficient interaction length and stable laser propagation is crucial for maximizing the role of electrons in the CSF and TRF.

## Methods

### Laser system

The experiments were carried out in the SULF laser facility^33^. It is a Ti: sapphire laser system with central wavelength λ = 800 nm. The laser beam is compressed by a four-grating compressor and the pulse duration is measured to be ~30 fs using a Fastlite Wizzler instrument. The energy transmission efficiency from the compressor to the target chamber is 67%. Double deformable mirrors are cascaded to optimize wavefront aberrations and, hence, the focal intensity. The *p*-polarized 2–PW laser was focused by an *f* / 2 off-axis parabolic mirror to a spot of 4.3 μm (1/*e*^2^ diameter), leading to a laser intensity of 5×10^21 ^W cm^–2^ with 56 J energy, corresponding to a relativistic normalized vector potential of *a*_0_ ~ 48. To improve temporal contrast, a combination of cross-polarized wave generation and a femtosecond optical parametric amplification technique is implemented at the front end, and in particular, a single PM was used in our experiments. The temporal pulse contrast without PM was measured to be 10^–11^ (10^–8^) at 100 (10) ps prior to the main pulse using a third-order autocorrelator.

### Diagnostics

A stack of image plates (IP, Fujifilm BAS-SR) and a metal filter with a 3mm-diameter hole were positioned 5cm behind the target, oriented at 5° relative to the normal and laser directions. Filters of varying thicknesses were placed in front of each IP to attenuate electron energy and shield protons. The iron plates between adjacent IPs have thicknesses of 1.8–3.2 mm, exhibit significantly stronger attenuation of electrons compared to background X/gamma rays. Monte Carlo simulations using Geant4 indicate that when the electrons irradiate a stack can result in bremsstrahlung, with photons above 100 keV readily escaping the filter plates. The IP BAS-SR shows a response to such X/gamma rays that is 1–3 orders of magnitude lower than to electrons. Each IP thus carries a weak, slowly decaying signal from these photons. The interference from photon signals is below 5%. The reference IP at the rear of the stack is used to help obtain the radiation noise. Subtracting the noise during the calculation of dN/dE of the spectrum, the impact of such radiation on electron measurements can be substantially reduced.

We mainly use a TPS to measure the proton energy spectra and determine the proton cutoff energy. It is composed of a 1.0 T magnetic field and equipped with 6 rotatable IP (Fujifilm BAS-TR) detectors^33^. The energy resolution is 1.3 MeV at 90 MeV and the lower energy threshold is 9 MeV. The TPS is placed at 90 cm behind the target, with a solid angle of 2.2 × 10^–8^ sr.

The THz measurement is shown in Fig. 1a. A plano-convex lens with a diameter of 2 inches and a focal length of 10 cm is placed at an angle of 30° to the normal behind the target (20° to the laser direction) to collect terahertz radiation. After passing through the lens, some terahertz waves are collimated and reflected by a gold mirror. They then pass through a polymethyl pentene (TPX) vacuum window and a filter (to block stray light signals) before entering the multi-channel terahertz spectrometer. The multi-channel THz spectrometer has been widely used for single-shot terahertz spectrum measurement in laser plasma experiments^34,52^. We use Si beam splitters to split the terahertz light into 8 beams, and each irradiates the pyroelectric detector (PD, SPY-01, Wired Sense) through a narrowband band-pass THz filter (BPF 0.1-15THz, Tydex) with a different central frequency. In order to obtain clean THz signals, the BPFs were attached on the PDs surfaces, and the spectrometer was adequately shielded from electromagnetic pulse and X-ray radiation. The energy of terahertz radiation is calculated from the PD voltage measured by the high-sampling-rate oscilloscope. Due to the introduction of a cutoff frequency of ~20 THz from TPX, we set the maximum measurement frequency band at 15 THz, and the central frequencies of the BPFs are 0.1, 0.3, 0.5, 1, 1.5, 3, 6, 10, and 15 THz. The entrance diameter of the terahertz spectrometer is 1 inch, which is much larger than the photosensitive area of the PD which is 4 mm^2^. The detector is calibrated to capture approximately 1.6×10^-4^ of the total radiation energy.

Due to spatial constraints behind the target, the THz spectrometer and electron IP stack were operated independently in sequential measurements. During THz measurements, the on-target laser energy was slightly elevated to 68 J. The spectral intensity is retrieved as *I*_THz_ = *V*_det_ / (*R*_det_ · *T* · Δω), where *V*_det_ is the detector signal, *R*_det_ is the detector responsivity, *T* is the overall transmittance of THz components in the path, and Δω is the transmission bandwidth of the BPF.

**Fig. 7:**
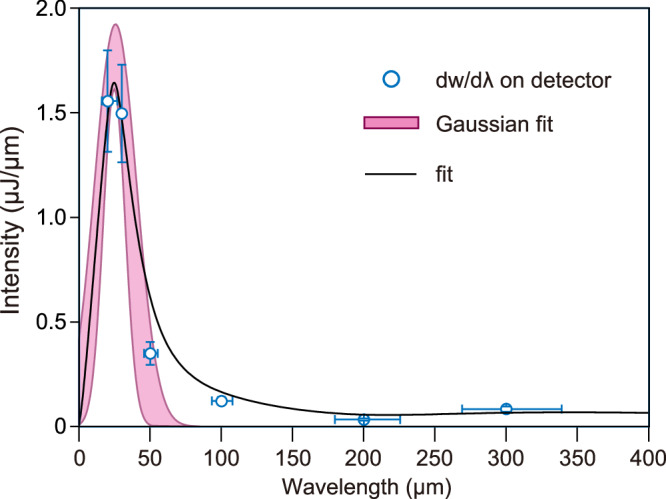
The measured THz wave spectrum. Each data point corresponds to the frequency data. The uncertainty in the duration is indicated by the red region.

To estimate the temporal duration of the measured THz radiation, we transformed the spectral profile from Fig. 3b into wavelength-dependent intensity (as shown in Fig. 7). Spectral analysis revealed a Gaussian distribution at short wavelength range (<100 μm), with a characteristic peak at *λ*_pk_ = 27 μm. The pulse duration was determined via the relation

3 $${\tau }_{{THz}}=({2\lambda }_{{pk}}-\bigtriangleup \lambda )/c$$

where *△λ* is the FWHM of wave spectrum.

### Simulations

The 3D Particle-In-Cell (PIC) simulations in the Simulation of TRF acceleration section are carried out using the Smilei code^53^. The simulation box size is 48 × 16 × 16 μm^3^ with cell size of 10 × 20 × 20 nm^3^ for the 30 μm CNF foam case. The box size is adjusted according to the CNF thickness. The electron density of the foam is 1 *n*_c_ and the target 500 *n*_c_, with 1 electron and 1 ion macroparticles per cell for the foam and 10 electrons and 10 ion macroparticles per cell for the substrate. A pre-plasma layer of *l*_pre_ = 0.1 μm is considered at the target rear surface. A *p*-polarized Gaussian laser pulse is incident vertically from the left boundary with a peak intensity of 5 × 10^21^ W cm^–2^. Low-density CH pollution with a thickness of 100nm was added to the rear of the SiN target, and it has a similar distribution profile to the substrate rear plasma.

To make direction comparison between theory and simulation results, the TRF from simulation must be extracted, which is difficult since the TRF overlaps with the longitudinal Coulomb fields. Therefore, we run a reference simulation of a depleted laser pulse interacting with the substrate (without foam) to obtain an approximation of the effective CSF and to reveal the contribution of the TRF. Here, the laser pulse is taken when it arrives at the substrate after depletion by the foam. This is done by dumping the entire electromagnetic field before the laser touches the substrate at 129fs, which is then injected into a simulation with only the substrate as the initial laser field. The CSF from this simulation is referred to as effective CSF *E*_eff.CSF_. This approach is employed under some approximations. For example, the TRF could be strong enough to deform the rear of the substrate. Second, the absence of foam removes the plasma channel effect within it. These effects are not included in the reference simulation without foam. Still, these effects do not undermine our conclusions.

The systematic validation of the TRF theoretical framework through 3D PIC simulations across various physical regimes, along with a detailed explanation of the post-processing method for decoupling the Coulomb and radiation fields, is provided in the Supplementary Information file.

To determine proton energies for foam thicknesses exceeding 60 μm, we further conduct 2D PIC simulations using the EPOCH code^54^. The simulation box size in *x* direction was adjusted according to the CNF thickness, and a 70 μm acceleration distance behind the substrate was ensured. All targets had 10° inclinations and were fully ionized and electrically neutral. For the CNF target, the electron density of the CNF and SiN layer were 1.0 *n*_*c*_ and 150 *n*_*c*_ (with preplasma) / 300 *n*_*c*_ (without preplasma), respectively. Exponential-distribution preplasma were considered only when CNF thickness <60 μm.

## Supplementary information

Supplementary Information

Transparent Peer Review file

## Data Availability

The experiment and simulation data generated in this study is freely available from the ScienceDB database under accession code 10.57760/sciencedb.32993 (ref. ^55^).

## Code availability

The open-source code Smilei is available at https://github.com/SmileiPIC/Smilei, and EPOCH is available at https://github.com/epochpic/epoch.
