# Supplementary material for: Transition Radiation Field Enhanced Laser Proton Acceleration Employing Near-Critical-Density Foam
Source: Nat Commun. 2026 Jun 15;17:7540. doi: 10.1038/s41467-026-74298-0 (PMC13409018; doi:10.1038/s41467-026-74298-0)
Supplement: Supplementary file 1 — Supplementary Information [file 41467_2026_74298_MOESM1_ESM.pdf]

Supplementary Information:

Transition Radiation Field Enhanced Laser Proton Acceleration Employing Near-Critical-Density Foam

C. Y. Qin[1,*], X. S. Geng[1,*], H. Zhang[1,2,†], L. H. Yu[1], L. G. Zhang[1], Y. Z. Dai[3], J. Wang[3], B. W. Zhang[1], X. J. Guo[1], D. R. Xu[1], S. Xu[1], C. L. Ding[3], Y. Xu[1], Y. Q. Liu[1,4], C. Wang[1], B. N. Shi[4], Z. X. Zhang[1], X. Y. Liu[1], Y. X. Leng[1,2], X. Y. Liang[1,2], B. F. Shen[5], L. L. Ji[1,2,‡] & R. X. Li[1,4,6,§]

[1]*State Key Laboratory of Ultra-intense Laser Science and Technology*, Chinese Academy of Sciences, Shanghai 20*1800, China*

[2]*CAS Center for Excellence in Ultra-intense Laser Science, Shanghai 201800, China*

[3]*Center of Materials Science and Optoelectronics Engineering, University of Chinese Academy of Sciences, Beijing 100049, China*

[4]*Zhangjiang Laboratory, Shanghai 201210, China*

[5]*Department of Physics, Shanghai Normal University, Shanghai 200234, China*

[6]*ShanghaiTech University, Shanghai 201210, China*

* These authors contributed equally.

† Email: zhanghui1989@siom.ac.cn

‡ E-mail: jill@siom.ac.cn

§ E-mail: ruxinli@mail.siom.ac.cn

I. Introduction

This supplementary document provides detailed theoretical treatment and comprehensive simulation analysis supporting the main manuscript on transition radiation field (TRF) enhanced laser proton acceleration. As discussed in the main text, the process of target normal sheath acceleration (TNSA) is often interpreted as the expansion of an electron-ion plasma in thermal equilibrium[1]. Hot electrons are much faster than ions, creating a charge-separation field (CSF) near the back surface (the sheath field) that accelerates ions. Meanwhile, some high-energy electrons are not confined by the sheath field and escape the target rear, generating transition radiation[2]. Therefore, during laser-foil interaction, both CSF and TRF coexist.

In the laser-foil case, since the number of high-energy electrons is relatively small, the TRF is generally weak, and its contribution to proton acceleration can be neglected. However, when near-critical-density (NCD) foam is attached to the foil, high-energy electron bunches are produced via direct laser acceleration (DLA)[3,4], and the number of collimated high-energy electrons significantly exceeds that from ponderomotive acceleration in TNSA. Consequently, transition radiation induced by DLA electrons becomes much stronger, leading to the enhanced proton acceleration observed experimentally.

In the following sections, we present the theoretical framework for separating the Coulomb field and TRF, followed by validation through three-dimensional particle-in-cell (PIC) simulations.

II. Theoretical framework

Field decomposition method. We consider a simplified model: a monoenergetic electron bunch passing through a plasma foil. In this case, the field of each electron consists of two components—the associated Coulomb field and the TRF generated upon crossing the interface. The former primarily corresponds to the CSF discussed in the main manuscript. While the Coulomb field co-moves with the electron, the TRF is radiated and decoupled from the electron, propagating at the speed of light.

For a relativistic electron, the Coulomb and TRF overlap with each other, making them difficult to decouple directly in PIC simulations. Therefore, we develop a post-processing method to analytically decompose each field based on the electron dynamics obtained from PIC simulations. By superimposing the derived fields of individual particles, collective fields of both types are revealed. This first-principle approach resolves CSF and TRF in laser-driven

plasma, where the former is consistent with that derived from Poisson's equation in the non- or weak relativistic regime.

Analytical solution for longitudinal electric field. Following the work by Carron[5], when an electron bunch exits the plasma boundary (considered as good conductors), the solution of the associated longitudinal electric field is given by:

$$E_x = \frac{Q}{\gamma^2}\left(\frac{1}{S_-} + \frac{1}{S_+}\right) - \frac{2Q}{R}, \quad 0 < T < \tau_{\text{beam}} \tag{1}$$

$$E_x = \frac{Q}{\gamma^2}\left(\frac{1}{S_-} - \frac{1}{S_{l-}} + \frac{1}{S_+} - \frac{1}{S_{l+}}\right), \quad T > \tau_{\text{beam}} \tag{2}$$

corresponding to retarded time within ($0 < T < \tau_{\text{beam}}$) and outside ($T > \tau_{\text{beam}}$) the pulse length. The parameters

$$S_{\pm} = \sqrt{(vt \pm x)^2 + \frac{r^2}{\gamma^2}} \tag{3}$$

account for the retarded positions of the beam and its image, capturing the finite propagation time of electromagnetic disturbances.

One finds that the term $-2Q/R$ in Eq. (1) represents the radiation field that decays as $1/R$ and extends to large distances. The terms with $1/\gamma^2$ prefactors describe the longitudinal components of the Coulomb fields, which are strongly suppressed for relativistic electrons. The transverse fields, on the other hand, are amplified by the relativistic effect.

In our modeling, we treat each macro particle in the simulation as a small bunch with length of the cell size. By summing up all the $-2Q/R$ terms and $1/\gamma^2$ terms of each macro particle, we obtain the collective radiation field and Coulomb field, respectively.

III. Validation through PIC simulations

To validate the theoretical framework, we systematically compare its predictions against 3D PIC simulations across distinct physical regimes, progressing from idealized to realistic electron bunch parameters.

Non-relativistic regime. We first examine a sub-relativistic electron bunch (Gaussian profile in three directions: $\sigma = 0.3\ \mu$m, density $n = 0.01n_c$) crossing a plasma-vacuum boundary with Lorentz factor $\gamma = 1.5$. The spatiotemporal evolution of the longitudinal electric field $\boldsymbol{E_x}$ is depicted in Fig. S1.

Apparently, the longitudinal Coulomb field, which co-propagates with the bunch, dominates the near-field structure and is much stronger than the TRF, as relativistic compression of the former is insignificant. Quantitatively, the peak Coulomb field reaches

approximately 1.5×10^{10} V/m, while the TRF amplitude remains 0.5×10^{10} V/m. As the bunch velocity is below c, the Coulomb field lags behind the emitted radiation. The theoretical predictions exhibit excellent agreement with the simulation results, demonstrating the accuracy of the modeling.

This Coulomb-dominated scenario is related to the CSF-type enhancement of sheath field, especially when sub- and near-relativistic electrons are the majority. The contribution of TRF on ion acceleration is negligible in this regime.

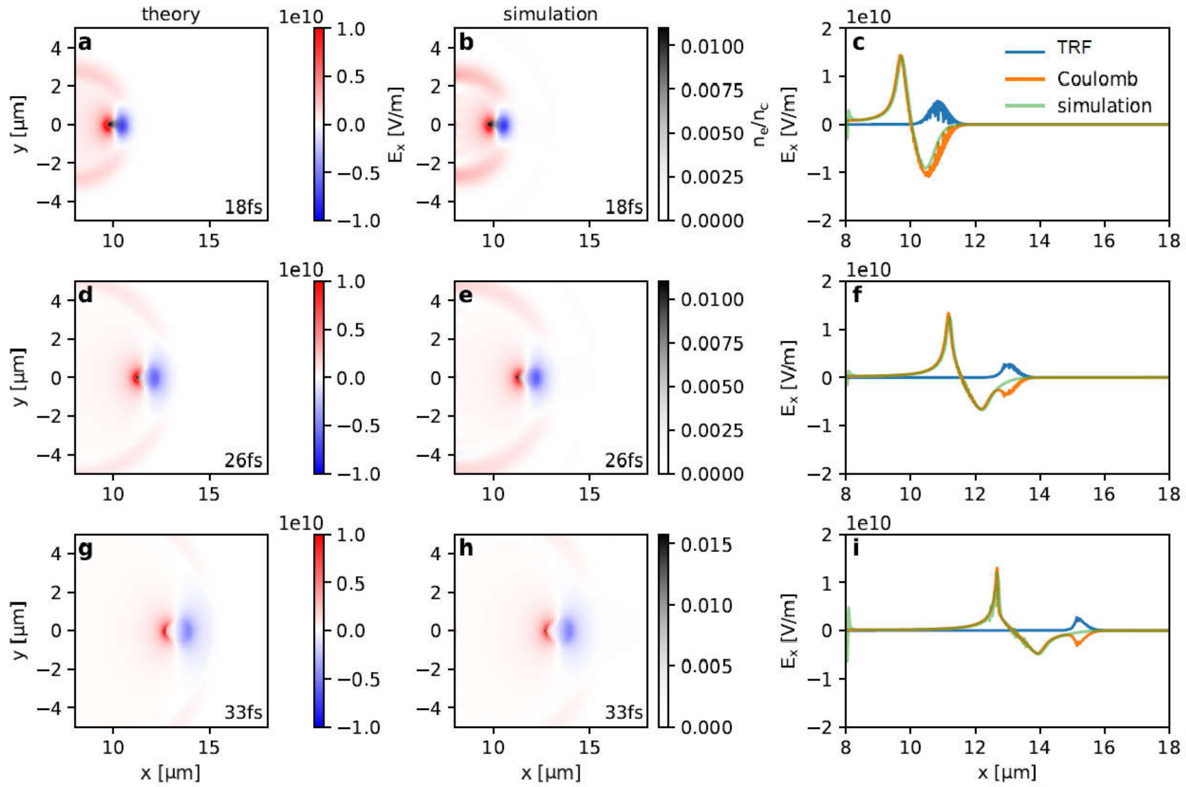

Fig. S1 Evolution of transition radiation field of a small electron bunch with $\gamma = 1.5$. a-c At 18 fs: **a** Calculated theoretical TRF, **b** 3D PIC simulation results, **c** On-axis acceleration field showing the calculated radiation term (TRF, blue), calculated Coulomb term (orange), and the simulation result (green). **d-f** At 26 fs. **g-i** At 33 fs.

Relativistic regime. Next, we increase the bunch energy to the relativistic regime ($\gamma = 10$). As shown in Fig. S2, the Coulomb field and TRF components co-propagate and cannot be directly decomposed in space.

We employ the post-processing model based on Eqs. (1-2), which perfectly reproduces the simulation results. In this regime ($\gamma = 10$), the longitudinal Coulomb field is suppressed by the factor, reducing from 1.5×10^{10} V/m to less than 10^9 V/m. Meanwhile, the TRF amplitude remains comparable levels of $\sim 2.5 \times 10^9$ V/m at the bunch position, making the

TRF contribution dominant. Consequently, the total accelerating field becomes a superposition of both contributions, with the TRF beginning to dominate the acceleration fields.

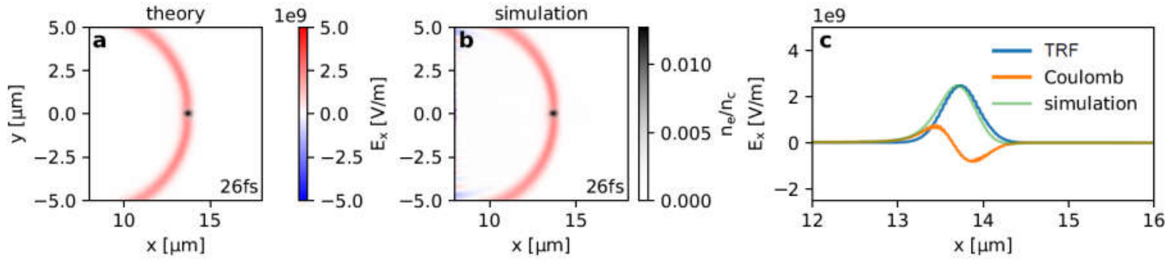

Fig. S2 Evolution of transition radiation field of a small electron bunch with $\gamma = 10$ at 26 fs. a Theoretical TRF. **b** Simulation results. **c** On-axis acceleration field of the calculated radiation term (TRF, blue), calculated Coulomb term (orange) and the simulation result (green).

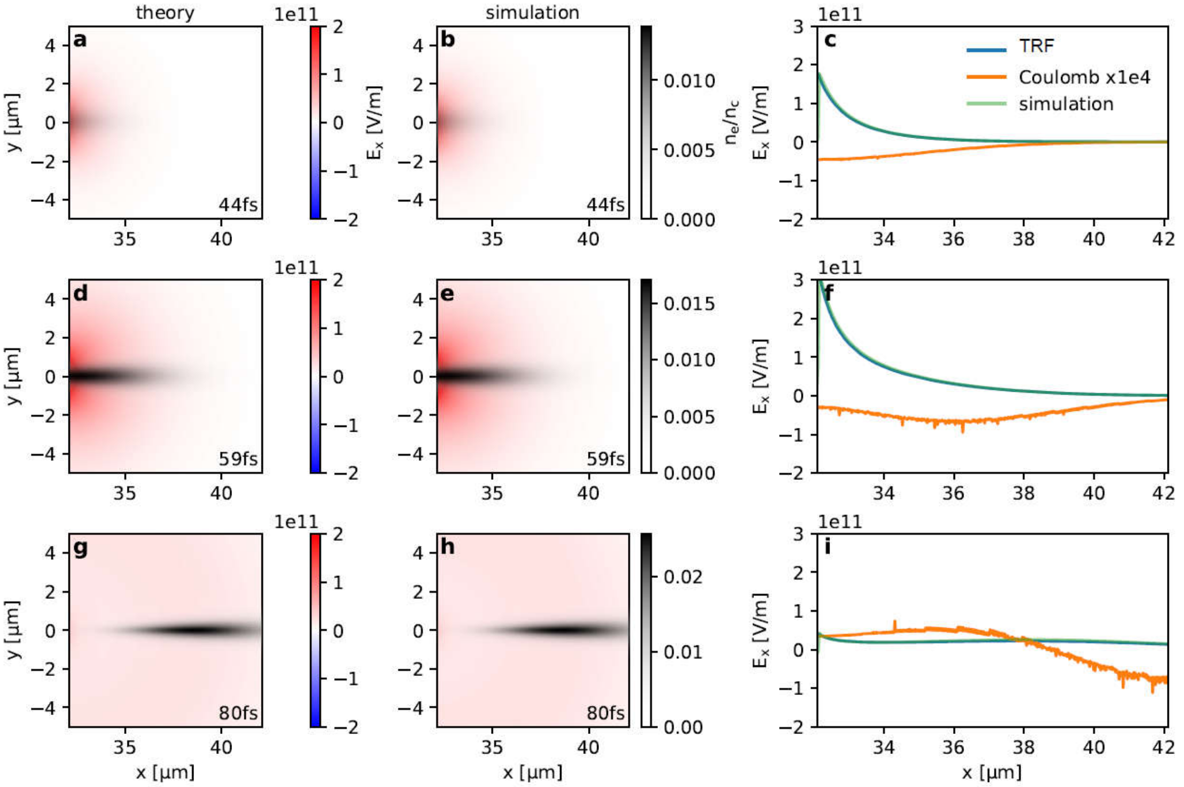

Fig. S3 Evolution of transition radiation field of a long bunch with $\gamma = 100$. a-c At 44 fs: **a** Theoretical TRF, **b** Simulation results, **c** On-axis acceleration field showing the calculated radiation term (TRF, blue), calculated Coulomb term (×10000, orange), and the simulation result (green). **d-f** At 59 fs. **g-i** At 80 fs.

Long bunch configuration. We then simulate a physically more accessible super-ponderomotive bunch characteristic of DLA mechanism (Gaussian profile: $5\ \mu\text{m} \times 1\ \mu\text{m} \times 1\ \mu\text{m}$, $n = 0.01n_c$ to suppress nonlinear effects, $\gamma = 100$). Again, the theoretical model matches well with the simulation, as shown in Fig. S3. Due to higher electron energies, the longitudinal Coulomb contribution becomes essentially negligible (amplified 10000 for visibility). The longitudinal accelerating field primarily arises from the TRF, reaching peak amplitudes approaching sub-TV/m levels (Figs. S3c, S3f). This electric field persists until the entire bunch vacates the target (Figs. S3g- S3i). In our experimental regime, the actual beam density may reach $\sim 0.1 - 1n_c$, thus capable of generating TRF of 1-10s' TV/m. Combined with its duration, TRF can have a significant impact on proton acceleration.

Wide bunch configuration. Finally, we investigate a transversely expanded bunch configuration ($1\ \mu m \times 5\ \mu m \times 5\ \mu m$ Gaussian). The above conclusions still hold for this case. Additionally, Fig. S4 demonstrates that this geometry sustains substantial transition radiation fields even after the bunch departs from the target, thereby significantly prolonging the effective acceleration duration compared to the elongated bunch case.

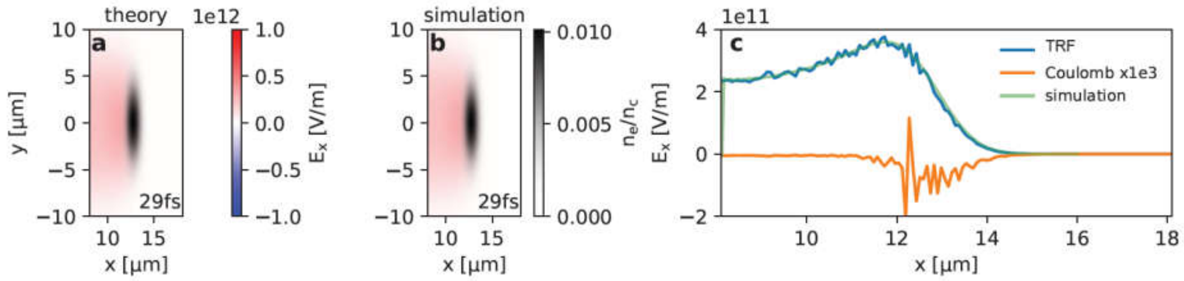

Fig. S4 Transition radiation field of a wide bunch with $\gamma = 100$ at 29 fs. a Theoretical TRF. **b** Simulation results. **c** On-axis acceleration field of the calculated radiation term (TRF, blue), calculated Coulomb term (×1000, orange) and the simulation result (green).

DLA bunch analysis. The above comparison of simulation and theory confirms the accuracy of our theory, indicating that in the plasma foil case, if the electron beam consists mainly of sub-/non-relativistic electrons, the longitudinal Coulomb field dominates; for strongly relativistic, collimated electron beams, the TRF dominates. We further apply our model to laser-plasma interactions in 3D PIC simulations with parameters similar to our experiment (see Fig. 4 of the main manuscript). The DLA bunches produced from NCD foam are injected into a thin substrate (energy spectrum is shown in Fig. S5a). Here the relativistic electrons are rather directional and of micro-bunching structures, extending both longitudinally and transversely. Comparing the theoretical results with simulation results, reasonable consistency can be seen in Fig. S5d, although not as perfect as the ideal cases above. From the comparison in Fig. S5d, it is evident that the TRF dominates in this case. The discrepancy between the theoretical post-processing and the PIC simulation is because we assume electron moving coaxial along x with the speed of light, which compromises the accuracy.

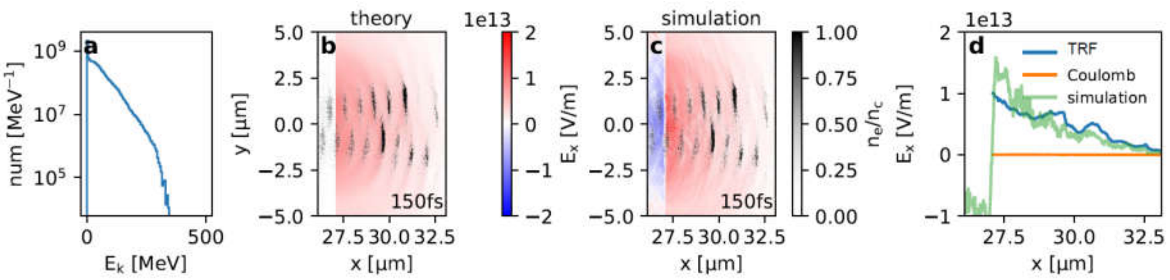

Fig. S5 Energy spectrum and transition radiation field of a DLA bunch traversing a foil at 150 fs. a Electron bunch energy spectrum. **b** Theoretical TRF. **c** Simulation results. **d** On-axis acceleration field of the calculated radiation term (TRF, blue), calculated Coulomb term (orange) and the simulation result (green). The simulation setup is identical to the manuscript with the laser removed.

Comparison to previous work. We now compare the above conclusions with previous work. For instance, the work of Bin et al[6]. acknowledged the “enhanced sheaths” effect induced by superponderomotive-electron (SPE) from NCD plasma. Yet, the qualitative narrative does not address the origin of such enhancement, *i.e.*, whether it is Coulomb type or something else. To address this, we carry out 3D simulations following the configuration from Ref. [6]. The original paper employed 2D simulations, while ours uses a full 3D geometry to check the origin of the “enhanced sheaths.” We calculate the longitudinal fields, both Coulomb field and TRF, in the same manner and compare this field in Fig. S6.

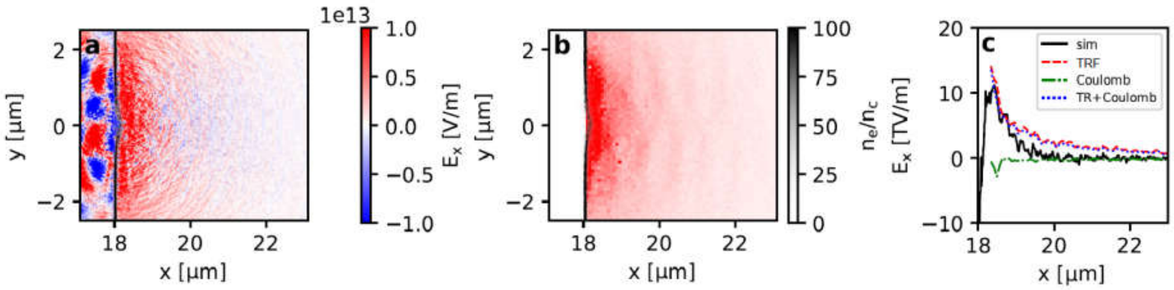

Fig. S6 Simulation comparison to the typical work. a Simulated longitudinal electric field E_x (red-blue) and electron density n_e (black-white) from 3D reconstruction of the configuration in Ref. [6]. **b** Theoretical calculation using Eqs. (1-2). **c** On-axis field comparison showing the simulation result (black-solid), theoretical radiation term (TRF, red-dashed), Coulomb term (green-dotted-dashed), and their superposition (blue-dotted).

It should be noted that SPEs exhibit large angular divergence, while our theoretical calculation assumes coaxial motion along the x-direction. This assumption could overestimate the TRF, yet the underlying physics remains clear. Our calculations show that the longitudinal Coulomb component is significantly lower than the TRF, as shown in Fig. S6c, consistent with previous relativistic cases. Based on these results, we conclude that the main contribution of SPEs to “enhanced sheaths” should be TRF in this case.

IV. Conclusion

The validation through PIC simulations from non-relativistic ($\gamma = 1.5$) to ultra-relativistic ($\gamma = 100$) electron energies, and from idealized Gaussian bunches to realistic DLA-produced electron distributions confirm the robustness of our theoretical approach. Furthermore, comparison with previous work demonstrates that the “enhanced sheath” effect observed in NCD foam experiments can be attributed primarily to transition radiation fields rather than Coulomb field enhancement, resolving a long-standing ambiguity in the interpretation of such experimental results.

Supplementary references

1. Mora, P. Plasma Expansion into a Vacuum. *Phys. Rev. Lett.* **90**, 185002 (2003).
2. Liao, G.-Q. *et al*. Towards Terawatt-Scale Spectrally Tunable Terahertz Pulses via Relativistic Laser-Foil Interactions. *Phys. Rev. X* **10**, 031062 (2020).
3. Pukhov, A., Sheng, Z.-M. & Meyer-ter-Vehn J. Particle acceleration in relativistic laser channels. *Phys. Plasmas* **6**, 2847–2854 (1999).
4. Hussein, A. E. *et al*. Towards the optimisation of direct laser acceleration. *New J. Phys.* **23**, 023031 (2021).
5. Carron, N. J. Fields of Particles and Beams Exiting a Conductor. *Prog. Electromagnetics Res.* **28**, 147-183 (2000).
6. Bin, J. *et al*. Enhanced Laser-Driven Ion Acceleration by Superponderomotive Electrons Generated from Near-Critical-Density Plasma. *Phys. Rev. Lett.* **120**, 074801 (2018).
