# Supplementary material for: Transition Radiation Field Enhanced Laser Proton Acceleration Employing Near-Critical-Density Foam
Source: Nat Commun. 2026 Jun 15;17:7540. doi: 10.1038/s41467-026-74298-0 (PMC13409018; doi:10.1038/s41467-026-74298-0)
Supplement: Supplementary file 2 — Transparent Peer Review file [file 41467_2026_74298_MOESM2_ESM.pdf]

nature portfolio

Peer Review File

Transition Radiation Field Enhanced Laser Proton Acceleration Employing Near-Critical-Density Foam

Corresponding Author: Professor Hui Zhang

This file contains all reviewer reports in order by version, followed by all author rebuttals in order by version.

Version 0:

Reviewer comments:

Reviewer #1

(Remarks to the Author)
The manuscript entitled "Transition Radiation Field Enhanced Laser Proton Acceleration Employing Near-Critical-Density Foam" presents an interesting experimental and numerical study of laser-driven proton acceleration where the authors report that transition radiation fields (TRFs), generated by energetic electron bunches exiting a near-critical-density (NCD) carbon nanotube foam (CNF) target, significantly enhance the maximum proton energy and reshape the proton spectrum. The work combines relativistically intense laser experiments at the state-of-the-art SULF facility with three-dimensional particle-in-cell (PIC) simulations to demonstrate that TRFs with strengths on the order of 10^12–10^13 V/m can superpose with the conventional target sheath field, doubling the proton cutoff energy to ~90 MeV and producing a plateau-shaped spectral feature. The authors argue that this hybrid acceleration scheme represents a new pathway for advancing laser-driven proton sources toward the >100 MeV energy range, which is higly demanded for applications in ion turmor therapy, compact accelerators, and high-energy-density science.

Briefly speaking, the topic is certainly timely and of potential broad interest to the readership of Nature Communications. Proton acceleration with high-power lasers remains a central goal of plasma-based acceleration research, with relevance to both fundamental physics and applications. In particular, the challenge of achieving proton energies exceeding 100 MeV in a stable, reproducible, and efficient manner is one of the major drivers of current international efforts. The authors propose to address this challenge by proposing an unconventional contribution from transition radiation fields, a phenomenon well-known in electron beam physics but rarely discussed in the context of laser ion acceleration. If substantiated, the claim that TRFs directly accelerate protons and lead to observable spectral signatures would represent a novel mechanism of significant conceptual interest.

While the manuscript presents an intriguing idea and a wealth of experimental and simulation data, I believe that several issues need to be addressed before it can be recommended for publication in Nature Communications. In what follows, I provide a detailed assessment of the manuscript's weaknesses, followed by extensive recommendations for revision. My comments are intended to be constructive, with the aim of helping the authors refine their presentation and clarify their claims.

1. Regarding the attribution of enhanced proton energies specifically to TRF, the manuscript makes a convincing case but could benefit from a more elaborative discussion. The observed doubling of proton cutoff energy when using ~35 um CNF is certainly consistent with the presence of an additional accelerating field beyond the standard sheath. However, other mechanisms might also contribute, such as stronger sheath fields generated by superponderomotive electrons, modified target rear expansion due to preplasma, or magnetic vortex structures formed in dense foam targets. While the authors argue that these mechanisms alone cannot explain the plateau-shaped proton spectrum, it would strengthen the paper if they explicitly discussed why TRF provides a more satisfactory explanation compared to these alternatives. Expanding the comparison would both highlight the novelty of their proposal and reassure readers that the TRF interpretation is not simply one of several possible stories.

2. The estimation of TRF strength from THz measurements deserves more detailed justification. The authors present convincing data showing sub-joule THz emission energies, correlated with electron charge, and they translate these into fields of 10^12–10^13 V/m. However, this translation relies on assumptions about source size, emission geometry, and temporal duration. The manuscript would benefit from a more transparent treatment of uncertainties. For example, the error

bars in Figure 3(a)(b) could be expanded to explicitly show measurement and calibration uncertainties. A brief sensitivity analysis of how variations in assumed beam diameter or pulse duration affect the estimated field would also add credibility. Importantly, the authors should emphasize that while exact field strengths may have uncertainty, the qualitative scaling with electron charge and foam thickness is robust, which is the key point for their argument.

3. The discussion of optimal foam thickness could be elaborated. The authors attribute the existence of an optimal thickness (~35 μm) to a trade-off between sufficient interaction length for electron acceleration and the onset of laser filamentation in thicker foams. This is a plausible explanation, but currently the evidence is limited to one simulation of laser channeling. More systematic simulation results could better support the argument. At minimum, the authors should include a clearer physical picture of how laser energy depletion and filamentation reduce electron collimation and, in turn, limit TRF generation. Adding a schematic or summary graph linking foam thickness to expected propagation regime would help readers understand why the optimum occurs where it does. Maybe it is better to show a large range of simulation domain from x=20 to x=40 um for the results in Figure 4.

4. The authors decouple TRF from the sheath field by running reference simulations without foam and subtracting the resulting sheath field from the total field in foam-included runs. This approach is reasonable, but the underlying assumptions should be better communicated. For instance, the linearity of superposition may not be perfect in a nonlinear plasma system. The authors should state explicitly that their method provides an approximate rather than exact separation of fields, and they should argue why it is nevertheless meaningful. Including numerical convergence tests, for example, showing that box size, resolution, or particle number do not qualitatively alter result, would also reassure readers. In addition, the phase-space plots of protons under TRF-enhanced versus sheath-only conditions are compelling; expanding these comparisons and linking them more directly to the experimental plateau spectrum would make the case for TRF much more persuasive.

5. Although the manuscript is well written, some passages could be clarified for a broader readership. The equations describing TRF fields (Eqs. 1–2) are mathematically correct but are introduced somewhat abruptly, with little physical explanation. Many readers of Nature Communications may not be familiar with transition radiation theory. The authors could improve accessibility by providing a short intuitive description, for example, explaining that TRF arises when a relativistic electron bunch crosses a plasma vacuum interface, generating coherent radiation because the bunch is much shorter than the radiation wavelength. Similarly, the experimental setup descriptions could be streamlined, focusing on the essential features rather than technical details of filters or detector models, which could be moved to Methods or Supplemetary information.

6. For applications, the manuscript mentions medical therapy and compact accelerators as motivations. While such aspirations are understandable, the current results remain below the energy thresholds (~150–200 MeV) required for clinical turmor therapy, and the plateau spectrum may not be optimal for narrow-bandwidth applications. The authors should moderate their claims, presenting applications as long term motivations rather than immediate prospects. Instead, they could emphasize the conceptual importance of discovering a new field contribution to proton acceleration, which may inspire further optimization. Such framing will strengthen credibility and avoid overstatement.

Overall, the manuscript presents a strong and potentially important contribution to the field of laser-driven ion acceleration. The experimental data are of high quality, the observed phenomena are interesting, and the proposed TRF-assisted mechanism is novel and worthy of attention. At the same time, the manuscript would benefit from revisions aimed at improving clarity, strengthening evidence, and moderating claims. These revisions do not require fundamentally new experiments, but rather deeper analysis, expanded discussion, clearer figures, and a more balanced presentation. I therefore recommend revision. With these improvements, the paper should be suitable for publication in Nature Communications and will attract wide interest among researchers in plasma physics, laser acceleration, and high-field science.

Minors:
Line 64: The work Ref. [31] shows that CNF can achieve a long acceleration time without sacrificing the strength of the acceleration field by utilizing composite targets. The strength of the accelerating electric field is not enhanced in the scenarios of Ref. [31].

Line 101: The proton energy spectrum in Figure 2(b) and 1(g) shows that the cutoff energy can approach 90 MeV, which is measured by using thomson parabola spectrometer (TPS). What about the experimental raw data of cutoff proton energy measured by the stack detector, such as CR-39 plates and radiochromic films (RCFs)?

Line 241: It might be helpful for understanding the acceleration mechanism by adding a subfigure to show the distribution of protons in the (x, px) phase space?

Line 283: Is the pulse duration of ~30 fs measured at the FWHM of the laser intensity? The abbreviation 'FWHM' is not defined in the manuscript.

Line 294: There should be some compatible problems. The sentence "Error! Reference source not found.a" does not make sense.

Line 276: It is better to set the white-green colorbar to be transparent for the part with 5 MeV to enhance the presentation.

Reviewer #2

(Remarks to the Author)
The manuscript by Qin et al reports proton cut-off energies up to ~90 MeV by irradiating PW Ti:Sa laser pulses onto near-critical density (NCD) foams layered on 100 nm SiN substrates. Varying foam thickness, they identify an optimum regime that yields a pronounced high-energy plateau in the proton spectra. The central interpretation is that, at this optimum, a high-charge relativistic electron bunch generates a coherent transition-radiation field (TRF) at the substrate rearside which overlaps and widens the rear-side accelerating region, thereby boosting proton energies and generating the high-energy plateau in the proton spectrum. For this optimum foam thickness, they report rear-emitted THz energies up to ~0.6 J across 0.1–15 THz and electron bunch charges up to ~26 nC. PIC simulations are used to argue that the TRF amplitudes can reach 10^{12} V/m, thereby significantly contributing to the proton acceleration.
The experimental dataset is interesting and showcases the current performance of their PW laser system with NCD foam-targets. However, in the context of today's state of the art, the reported ~90 MeV from 56 J is not, by itself, a high-impact outlier: recent experiments have reached ~150 MeV with ~22 J via cascaded transparency regimes [Ziegler et al., Nature Physics (2024] and >110 MeV using conduction-restricted nanometre foils [Shou et al., Nat. Commun. (2025)]. NCD foams and double-layer targets are already established routes for boosting electron and ion energies, with multiple experiments attributing the gains chiefly to superponderomotive electrons that strengthen the sheath fields [Bin et al., PRL (2018); Ma et al. PRL (2019)]. Intense X-ray emission from foams and the use of THz diagnostics to probe ultrafast laser–target dynamics are likewise published (e.g. [Shou et al., Opt. Lett. (2021); Tavana et al., Frontiers in Physics (2023); Liao et al., PRL (2024)]). What the authors claim to be new in their manuscript is the interpretation that a coherent TRF contribution enhances the proton acceleration. The paper's impact therefore hinges on convincingly establishing that TRF contribution beyond reasonable alternatives.
At present, the data and analysis do not convincingly support their central claim that TRF is a decisive, distinct contributor to acceleration beyond a superponderomotive electron enhanced sheath. The most important issues are:
1) Shot-to-shot stability and causality.
THz, electron, and proton data were not recorded on the same shots and, critically, were taken at different laser energies (≈56 J for ions/electrons vs ≈68 J for THz—>20% higher), so the reported correlations are indirect. Please also clarify whether transmitted 800 nm light was detected behind the 100 nm substrate; at the stated peak intensities partial transparency is plausible and would affect the rear conditions. In addition, the total number of shots per setting and precise error-bar definitions are missing in Figs. 2–3; mean values can be misleading when the total number of shots is small.

2) Quality of experimental data:
The electron IP stack at 5 cm is highly vulnerable to contamination by X-/γ-rays from the foam/target and bremsstrahlung from ions/electrons in upstream absorbers. Electron specificity has not been demonstrated. Established controls—photon/electron discrimination using additional magnetic deflection and shielding, plus Monte-Carlo dose estimates for the exact geometry—are needed, with the resulting systematics propagated into any correlations. For the THz diagnostics, a complete calibration is required: full optical transfer function and geometric acceptance with uncertainties, and an independent energy/time cross-check. The pulse-duration estimate derived from channelized spectra is heuristic and should be justified or replaced by a defensible time-domain retrieval within the stated bandwidth limits.

3) Mechanism ambiguity:
There is substantial ambiguity with respect to established explanations—most notably superponderomotive-electron (SPE) enhanced sheaths in double-layer/NCD targets [Bin et al., PRL (2018); Ma et al. PRL (2019)] and magnetic-vortex acceleration (MVA) in near-critical channels. These prior experiments with closely related target concepts described the enhanced acceleration performance to these electrons that amplify the rear sheath; MVA and surface-wave currents can also contribute when long NCD channels and strong azimuthal fields form. In the present manuscript, a systematic classification and delimitation from these mechanisms is missing. The data do not yet show which part of the observed enhancement and plateau uniquely requires a TRF contribution—versus being explainable by an SPE-dominated sheath or by MVA inside the foam. I do appreciate the general simulation strategy—running a full foam case and a separate "effective sheath" scenario and then comparing to an analytic TRF model—but, as presented, the decomposition is not yet convincing. In particular, the Coulomb (space-charge) fields of the high-charge electron bunches can contribute substantially and are not cleanly separated from the purported radiative TR component. Further discriminating signatures might be available in the authors hypothesis but are not provided. Without discussing and discriminating from alternative interpretations and mechanisms the entire novelty about the TRF contribution remains ambiguous
Until these issues are addressed, I cannot recommend publication in its present form. The paper may ultimately be stronger if it does not hinge on proving a single "new" mechanism. Reframing the study as a careful, quantitative exploration of how SPE-enhanced sheaths, transition-radiation fields, and possible MVA/surface currents interplay in the NCD-foam regime—supported by targeted discriminators—could make a valuable contribution even without asserting one dominant mechanism.

Version 2:

Reviewer comments:

Reviewer #1

(Remarks to the Author)
Dear Editor,

I have carefully read the revised manuscript together with the authors' detailed point-by-point responses to the referees' comments. I appreciate the considerable effort the authors have made to address the concerns raised in the first round of review. In my opinion, the responses are thorough and constructive, and the revised manuscript has been clearly improved

in terms of clarity, physical interpretation, and supporting analysis.

As said in my previous report, this work represents a potentially strong and important contribution to the field of ion acceleration driven by relativistic laser–plasma interactions. The experimental observations are compelling, and the combination of experimental measurements with three-dimensional PIC simulations provides a solid and comprehensive basis for the conclusions. In particular, the proposed transition radiation field (TRF) assisted acceleration mechanism offers a novel and conceptually interesting explanation for the enhanced longitudinal electric field responsible for the observed proton acceleration.

In conventional studies of laser-driven proton acceleration, the longitudinal accelerating field is typically attributed to sheath fields generated by charge separation at the rear surface of the target, as described in the target normal sheath acceleration (TNSA) framework. In contrast, the present work proposes that transition radiation fields produced by relativistic electron bunches exiting the plasma can significantly contribute to, and even dominate, the accelerating field. This interpretation provides a new perspective on how relativistic electron dynamics may influence the acceleration environment, particularly in near-critical density targets where superponderomotive electrons are generated.

In the revised manuscript, the authors have provided substantially expanded analysis to support this interpretation. They introduce a theoretical framework that separates the longitudinal electric field into Coulomb and transition-radiation components based on the dynamics of relativistic electron bunches. The comparison between this analytical treatment and 3D PIC simulation results strengthens the argument that the transition radiation field can play a dominant role under the conditions considered. In addition, the authors have also clarified several issues raised in the first review, including the THz-based estimation of the TRF amplitude and its uncertainties, the discussion of optimal foam thickness, and the assumptions used to distinguish TRF contributions from conventional sheath fields in the simulations. These revisions improve the overall clarity and balance of the manuscript.

Overall, my previous concerns have been satisfactorily addressed. I therefore believe the manuscript now meets the standards of Nature Communications and strongly recommend its publication.

With best regards,
Zheng Gong

Reviewer #2

(Remarks to the Author)
I appreciate that the authors have made a serious and constructive effort to address the concerns raised by both reviewers. In particular, it is clear that the ambiguity of the acceleration mechanism was one of the main weaknesses of the original manuscript, and the revised version responds to this issue in a much more substantial way. The added theoretical treatment and simulation analysis, especially the simplified scenario of a relativistic electron bunch traversing a plasma foil to separate Coulomb and radiative contributions, are compelling and significantly strengthen the paper.
These additions are valuable beyond the immediate manuscript, and the authors could consider retaining the key arguments in concise form in the main text while moving the more extended derivations, discussion, and supporting figures to a Supplementary Information file. This would help keep the manuscript focused and readable while still making the full analysis accessible to interested readers.
The authors are also right that, in earlier studies of superponderomotive-electron–enhanced proton acceleration, the microscopic coupling between the electrons and the accelerating fields was often discussed only briefly. If the interpretation advanced here, that a significant part of what has previously been described as an “enhanced sheath” can in fact be understood more specifically in terms of transition-radiation fields generated by relativistic electrons, proves to be more generally valid, this would be an important observation for the community. In that sense, the manuscript has the potential to stimulate useful discussion and motivate more targeted experiments in the future.
At the same time, some of the experimental correlations remain indirect, and ambiguities with respect to alternative or concurrent mechanisms persist. I therefore do not regard the present data as a definitive demonstration that TRF is the dominant mechanism in this specific case. That said, the level of evidence is broadly comparable to what has been accepted in other published studies of similar scenarios. Two points nevertheless deserve further attention. First, the statistical presentation of the experimental data should be reconsidered: standard deviations derived from only two or three shots are of limited significance, and it would be more informative to show the individual shots directly rather than only mean values with error bars. Second, it should be stated clearly in the main text, not only in the caption of Fig. 3, that the electron and terahertz signals were diagnosed independently in separate measurements and at different laser energies.
On balance, the revised manuscript now presents an interesting and physically well-motivated interpretation, supported by a much stronger theoretical and numerical framework, and I believe it merits publication and discussion by the community, provided the authors present their central claim with appropriate caution and do not overstate the exclusivity of the mechanism.

Open Access This Peer Review File is licensed under a Creative Commons Attribution 4.0 International License, which permits use, sharing, adaptation, distribution and reproduction in any medium or format, as long as you give appropriate credit to the original author(s) and the source, provide a link to the Creative Commons license, and indicate if changes were made.
In cases where reviewers are anonymous, credit should be given to 'Anonymous Referee' and the source.
The images or other third party material in this Peer Review File are included in the article's Creative Commons license, unless indicated otherwise in a credit line to the material. If material is not included in the article's Creative Commons license and your intended use is not permitted by statutory regulation or exceeds the permitted use, you will need to obtain permission directly from the copyright holder.
To view a copy of this license, visit https://creativecommons.org/licenses/by/4.0/

Point-to-point responses to Reviewers' comments

Dear Reviewers,

We greatly appreciate your efforts in reviewing our manuscript. After carefully reading the reports, we think your comments and suggestions are very pertinent. In the following, we would like to answer the questions you mentioned and give detailed account of the changes made to the original manuscript. The corresponding revision in our manuscript is marked in red.

Reviewer #1

General comment: The manuscript entitled "Transition Radiation Field Enhanced Laser Proton Acceleration Employing Near-Critical-Density Foam" presents an interesting experimental and numerical study of laser-driven proton acceleration where the authors report that transition radiation fields (TRFs), generated by energetic electron bunches exiting a near-critical-density (NCD) carbon nanotube foam (CNF) target, significantly enhance the maximum proton energy and reshape the proton spectrum. The work combines relativistically intense laser experiments at the state-of-the-art SULF facility with three-dimensional particle-in-cell (PIC) simulations to demonstrate that TRFs with strengths on the order of 10^12–10^13 V/m can superpose with the conventional target sheath field, doubling the proton cutoff energy to ~90 MeV and producing a plateau-shaped spectral feature. The authors argue that this hybrid acceleration scheme represents a new pathway for advancing laser-driven proton sources toward the >100 MeV energy range, which is highly demanded for applications in ion tumor therapy, compact accelerators, and high-energy-density science.

Briefly speaking, the topic is certainly timely and of potential broad interest to the readership of Nature Communications. Proton acceleration with high-power lasers remains a central goal of plasma-based acceleration research, with relevance to both fundamental physics and applications. In particular, the challenge of achieving proton energies exceeding 100 MeV in a stable, reproducible, and efficient manner is one of the major drivers of current

international efforts. The authors propose to address this challenge by proposing an unconventional contribution from transition radiation fields, a phenomenon well-known in electron beam physics but rarely discussed in the context of laser ion acceleration. If substantiated, the claim that TRFs directly accelerate protons and lead to observable spectral signatures would represent a novel mechanism of significant conceptual interest.

While the manuscript presents an intriguing idea and a wealth of experimental and simulation data, I believe that several issues need to be addressed before it can be recommended for publication in Nature Communications. In what follows, I provide a detailed assessment of the manuscript's weaknesses, followed by extensive recommendations for revision. My comments are intended to be constructive, with the aim of helping the authors refine their presentation and clarify their claims.

Overall, the manuscript presents a strong and potentially important contribution to the field of laser-driven ion acceleration. The experimental data are of high quality, the observed phenomena are interesting, and the proposed TRF-assisted mechanism is novel and worthy of attention. At the same time, the manuscript would benefit from revisions aimed at improving clarity, strengthening evidence, and moderating claims. These revisions do not require fundamentally new experiments, but rather deeper analysis, expanded discussion, clearer figures, and a more balanced presentation. I therefore recommend revision. With these improvements, the paper should be suitable for publication in Nature Communications and will attract wide interest among researchers in plasma physics, laser acceleration, and high-field science.

Response: We are really appreciated of your clear and precise summary on the highlights and innovations of our study, and grateful for the careful review and thoughtful comments that helped us significantly improve our manuscript.

Comment 1: Regarding the attribution of enhanced proton energies specifically to TRF, the manuscript makes a convincing case but could benefit from a more elaborative discussion. The observed doubling of proton cutoff energy when using ~35 um CNF is certainly consistent with the presence of an additional accelerating field beyond the standard sheath. However, other mechanisms might also contribute, such as stronger sheath fields generated

by superponderomotive electrons, modified target rear expansion due to preplasma, or magnetic vortex structures formed in dense foam targets. While the authors argue that these mechanisms alone cannot explain the plateau-shaped proton spectrum, it would strengthen the paper if they explicitly discussed why TRF provides a more satisfactory explanation compared to these alternatives. Expanding the comparison would both highlight the novelty of their proposal and reassure readers that the TRF interpretation is not simply one of several possible stories.

Response:

According to the Reviewer's suggestion, we elaborate the enhanced acceleration via transition radiation field (TRF) in the following. Some of the discussion is also further stressed in the response to your other comments.

The process of TNSA is often interpreted as the expansion of an electron-ion plasma in thermal equilibrium [P. Mora, Phys. Rev. Lett. 90, 185002 (2003)]. The hot electrons are much faster than the ions, therefore creating charge-separation field (CSF) near the back surface, i.e., the sheath field, accelerating the ions. Electrons can also get reflected by the sheath field, leading to recirculation and hence optimized target thickness [Sentoku et al., Phys. Plasmas 10, 2009 (2003)]. Meanwhile, some electrons of high energies are not confined by the sheath field, and escape the target rear, generating transition radiation [Liao, G.-Q. et al. Phys. Rev. X 10, 031062 (2020)]. Therefore, during the laser-foil interaction, both the CSF and the TRF coexist. In the laser-foil case, however, since the number of high-energy electrons is relatively small (will be discussed below), the TRF is generally weak, and its contribution to proton acceleration can be neglected.

When the NCD foam is attached to the foil, high-energy electron bunches are produced via direct-laser-acceleration (DLA) [Pukhov, A., et al. Physics of Plasmas 6, 2847–2854 (1999). Hussein, A. E. et al. New J. Phys. 23, 023031 (2021).], and the number of collimated high-energy electrons significantly exceeds that from ponderomotive acceleration in TNSA. Consequently, transition radiation induced by DLA electrons are getting much stronger. We will analyze the fields generated by these high-energy electrons and their contributions to proton acceleration from the following aspects.

We consider a simplified model: a monoenergetic electron bunch passing through a

plasma foil. In this case, the field of each electron consists of two components—the associated Coulomb field and the TRF generated upon crossing the interface. The former primarily corresponds to the CSF. While the Coulomb field comoves with the electron, the TRF is radiated and decoupled from the electron. For a relativistic electron, the Coulomb and TRF overlap with each other - difficult to decouple in PIC simulations. Therefore, we develop a post-processing method to analytically decompose each field based on the electron dynamics obtained from PIC simulations. By superimposing the derived fields of individual particles, collective fields of both types are revealed. This first-of-principle approach resolves CSF and TRF in laser-driven plasma, where the former is consistent with that derived from Poisson's equation in the non- or weak relativistic regime.

Following the work by Carron, N. J. [Carron, PIER 28, 147-183 (2000)], when an electron bunch exits the plasma boundary (considered as good conductors), the solution of the associate longitudinal electric field is (The Eqs. (1-2) in the manuscript and Eqs. (56-57) of [Carron, PIER 28, 147-183 (2000)])

$$E_x = \frac{Q}{\gamma^2}\left(\frac{1}{S_-} + \frac{1}{S_+}\right) - \frac{2Q}{R},\ 0 < T < \tau_{beam} \quad (1)$$

$$E_x = \frac{Q}{\gamma^2}\left(\frac{1}{S_-} - \frac{1}{S_{l_-}} + \frac{1}{S_+} - \frac{1}{S_{l_+}}\right),\ T > \tau_{beam}. \quad (2)$$

corresponding to retarded time within ($0 < T < \tau_{beam}$) and outside ($T > \tau_{beam}$) the pulse length. The parameters $S_\pm = \sqrt{(vt \pm x)^2 + \frac{r^2}{\gamma^2}}$ account for the retarded positions of the beam and its image, capturing the finite propagation time of electromagnetic disturbances. One finds that the term −2Q/R in Eq. (1) represents the radiation field that decays as 1/R and extends to large distance. The terms with $1/\gamma^2$ prefactors describe the longitudinal components of the Coulomb fields, which are strongly suppressed for relativistic electrons. The transverse fields, on the other hand, is amplified by the relativistic effect. In our modelling, we treat each macro particle in the simulation as a small bunch with length of the cell size. By summing up all the −2Q/R term and $1/\gamma^2$ terms of each macro particle, we get the collective radiation field and Coulomb field.

1. Calibration of the theory with Simulations

To validate the theoretical framework, we systematically compare its predictions against 3D

PIC simulations across distinct physical regimes, progressing from idealized to realistic electron bunch parameters.

Non-relativistic regime

We first examine a sub-relativistic electron bunch (Gaussian profile in three directions: $\sigma = 0.3$ μm, density $n = 0.01n_c$) crossing a plasma-vacuum boundary with Lorentz factor $\gamma = 1.5$. The spatiotemporal evolution of the longitudinal electric field E_x is depicted in Fig. R1. Apparently, the longitudinal Coulomb field, which co-propagates with the bunch, dominates the near-field structure and is much stronger than the TRF, as relativistic compression of the former is insignificant. As the bunch velocity is below c, the Coulomb field lags the emitted radiation. The theoretical predictions exhibit excellent agreement with the simulation results, demonstrating the accuracy of the modelling. This Coulomb-dominated scenario is related to the CSF-type enhancement of sheath field, especially when sub- and near-relativistic electrons are the majority. The contribution of TRF on ion acceleration is negligible.

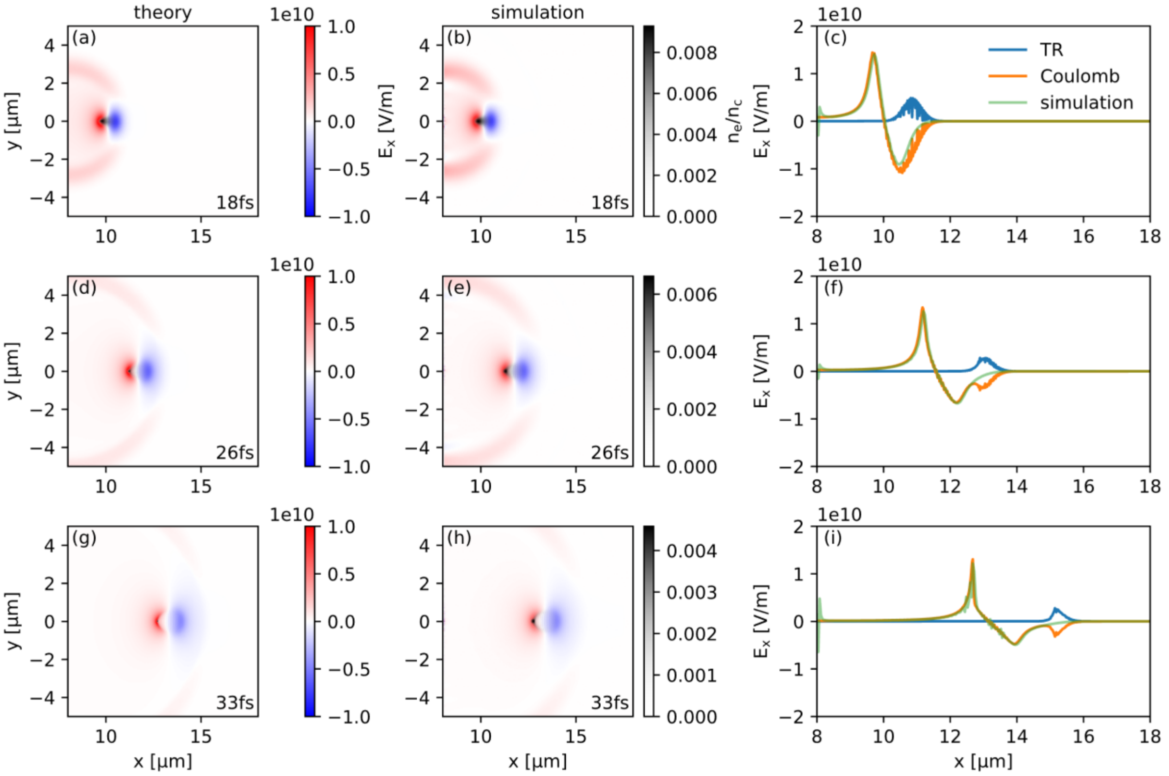

Figure R1. Evolution of transition radiation field of a small electron bunch with $\gamma = 1.5$. (a) Calculated theoretical TR field. (b) 3D PIC Simulation results. (c) On-axis acceleration field of the calculated radiation term (TR, blue), calculated Coulomb term (orange) and the

simulation result (green). (a-c) At 18fs. (d-f) At 26fs. (d-f) At 33s.

Relativistic regime

Next, we increase the bunch energy to relativistic regime ($\gamma = 10$). As one can see in Fig. R2, the Coulomb and TR components co-propagate and cannot be directly decomposed in space. We employe the post-processing model based on Eqs. (1-2), which perfectly reproduces the simulation results. As shown in Fig. R2 (c), relativistic effect significantly suppresses the longitudinal Coulomb component and TRF (extracted from simulation data and post-processed by Eqs. (1) and (2)) becomes dominant. In other words, the contribution of TRF surpasses that of Coulomb part when electrons are relativistic. Here we consider a small bunch; next we will examine longer and wider electron bunches. We will see that although TRF of each electron propagates at the speed of light, the duration and extended region of collective TRF is dependent on the size of the whole electron source, thereby enabling sustaining acceleration for considerable time and distance.

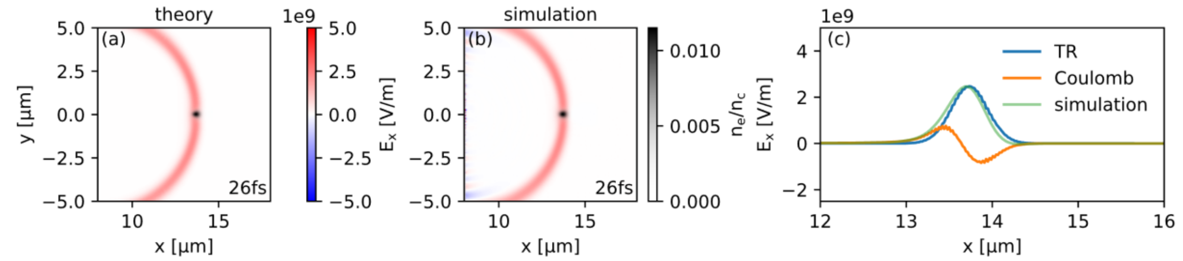

Figure R2. Evolution of transition radiation field of a small electron bunch with $\gamma = 10$ at 26 fs. (a) Theoretical TR field. (b) Simulation results. (c) On-axis acceleration field of the calculated radiation term (TR, blue), calculated Coulomb term (orange) and the simulation result (green).

Long-bunch configuration

We then simulate a physically more accessible superponderomotive bunch characteristic of DLA mechanism (Gaussian profile: 5 μm × 1 μm × 1 μm, $n = 0.01n_c$ to suppress nonlinear effects, γ = 100). Again, the theoretical model matches well with the simulation, as shown in Fig. R3. Due to higher electron energies, the longitudinal Coulomb contribution becomes essentially negligible (amplified 10000 for visibility). The longitudinal accelerating field primarily arises from the TRF, reaching peak amplitudes approaching sub-

TV/m levels (Figs. long-case(c) & (f)). This electric field persists until the entire bunch vacates the target (Figs. long-case(g-i)). In our experimental regime, the actual beam density may reach $\sim 0.1 - 1n_c$, thus capable of generating TRF of 1-10s' TV/m. Combined with its duration, TRF can have a significant impact on proton acceleration.

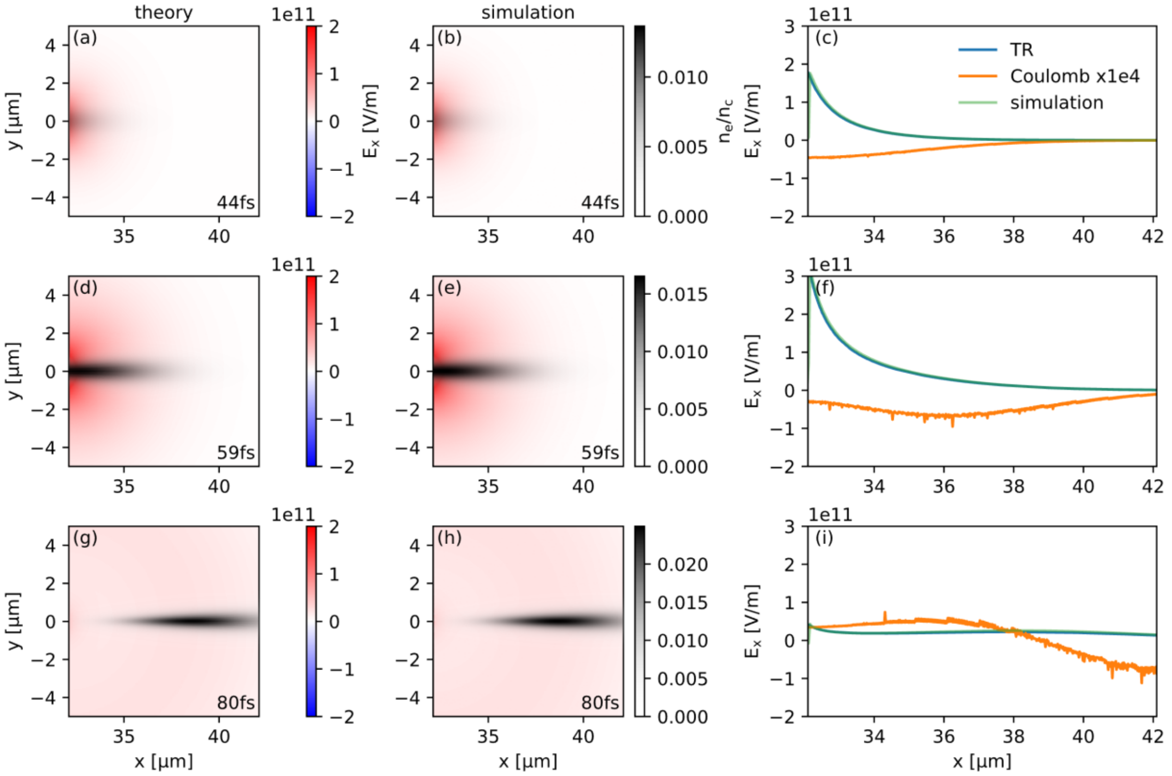

Figure R3. Evolution of transition radiation field of a long bunch with $\gamma = 100$. (a) Theoretical TR field. (b) Simulation results. (c) On-axis acceleration field of the calculated radiation term (TR, blue), calculated Coulomb term (×10000, orange) and the simulation result (green). (a-c) At 44fs. (d-f) At 59fs. (d-f) At 80fs.

Wide-bunch configuration

Finally, we investigate a transversely expanded bunch configuration (1 μm × 5 μm × 5 μm Gaussian). The above conclusions still hold for this case. Additionally, Fig. R4 demonstrates that this geometry sustains substantial transition radiation fields even after the bunch departs from the target, thereby significantly prolonging the effective acceleration duration compared to the elongated bunch case.

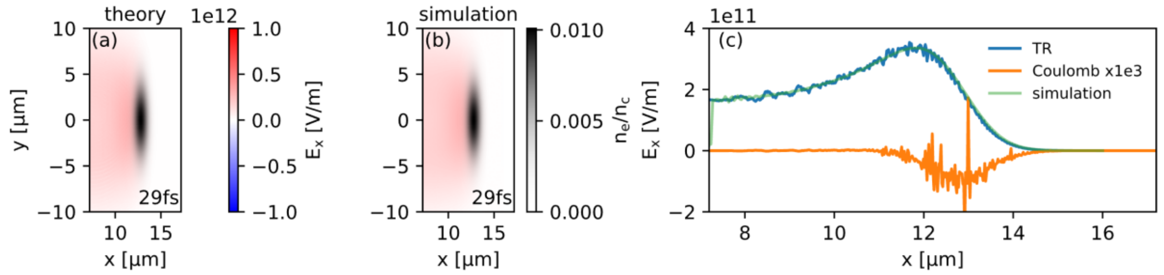

Figure R4. Transition radiation field of a wide bunch with $\gamma = 100$ at 29 fs. (a) Theoretical TR field. (b) Simulation results. (c) On-axis acceleration field of the calculated radiation term (TR, blue), calculated Coulomb term (×1000, orange) and the simulation result (green).

2. Longitudinal Coulomb field and TRF in laser-plasma interaction

The above comparison of simulation and theory confirms the accuracy of our theory, indicating that in the plasma foil case, if the electron beam consists mainly of sub-/non-relativistic electrons, the longitudinal Coulomb field dominates; for strongly relativistic, collimated electron beams, the TRF dominates. We further apply our model to laser-plasma interactions in 3D PIC simulations with parameters similar to our experiment. The DLA bunches produced from NCD foam are injected into a thin substrate (energy spectrum is shown in Fig. R5 (a)). Here the relativistic electrons are rather directional and of micro-bunching structures, extending both longitudinally and transversely. Comparing the theoretical results with simulation results, reasonable consistency can be seen in Fig. R5 (d), although not as perfect as the ideal cases above. From the comparison in Fig. R5 (d), it is evident that the TR dominates in this case. The discrepancy between the theoretical post-processing and the PIC simulation is because we assume electron moving coaxial along x with the speed of light, which compromises the accuracy.

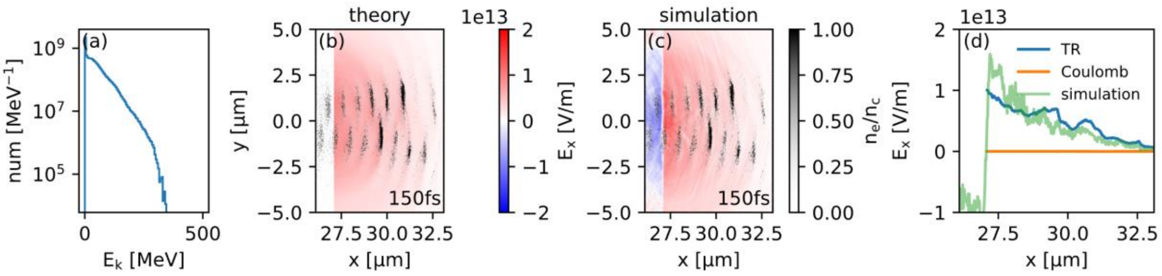

Figure R5. Energy spectrum and transition radiation field of a DLA bunch traversing a foil at 150 fs. (a) Electron bunch energy spectrum. (b) Theoretical TR field. (c) Simulation

results. (d) On-axis acceleration field of the calculated radiation term (TR, blue), calculated Coulomb term (orange) and the simulation result (green). The simulation setup is identical to the manuscript (Fig. 4) with the laser removed.

3. Comparison to previous work

We now compare the above conclusions with previous work. For instance, the work of Bin et al. [Phys. Rev. Lett. 120, 074801 (2018)] acknowledged the "enhanced sheaths" effect induced by superponderomotive-electron (SPE) from NCD plasma. Yet, the qualitative narrative does not address the origin of such enhancement, i.e., whether it is Coulomb type or something else. To address this, we carry out 3D simulations following the configuration from Ref. [Bin et al., Phys. Rev. Lett. 120, 074801 (2018)]. The original paper employed 2D simulations, while ours uses a full 3D geometry to check the origin of the "enhanced sheaths." We calculate the longitudinal fields, both Coulomb and TR, in the same manner and compare this field in Fig. R6.

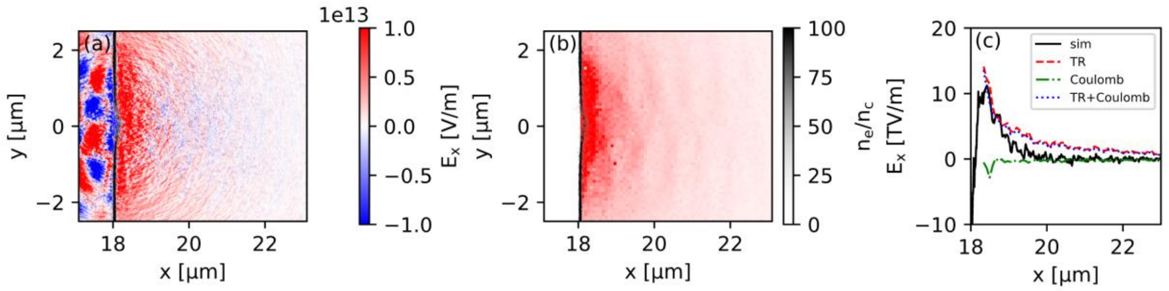

Figure R6. (a) Simulated longitudinal electric field E_x (red-blue) and electron density n_e (black-white) from 3D reconstruction of the configuration in Ref. [Bin et al., Phys. Rev. Lett. 120, 074801 (2018)]. (b) Theoretical calculation using Eqs. (1-2). (c) On-axis field comparison showing the simulation result (black-solid), theoretical radiation term (TR, red-dashed), Coulomb term (green-dotted-dashed), and their superposition (blue-dotted).

It should be noted that SPEs exhibit large angular divergence, while our theoretical calculation assumes coaxial motion along the x-direction. This assumption could overestimate the TR fields, yet the underlying physics remains clear. Our calculations show that the longitudinal Coulomb component is significantly lower than the TRF, as shown in

Fig. R6 (c), consistent with previous relativistic cases. Based on these results, we conclude that the main contribution of SPEs to "enhanced sheaths" should be TRF in this case.

4. Comparison to Magnetic vortex acceleration

In terms of magnetic vortex acceleration (MVA), the proposal by Nakamura et al. [Phys. Rev. Lett. 105, 135002 (2010)] showed that expansion of magnetic field within plasma channels induces strong longitudinal electric field, as shown in Fig. R7. The main difference in our configuration is that there is a high-density SiN substrate of $500n_c$ and 0.1μm thickness attached to the NCD foam. No laser penetration is observed in both our experiments and PIC simulations. Therefore, the magnetic fields generated within the foam is largely blocked by the substrate. As shown in Fig. R8 (a), the distribution of the vortex field B_z is discontinuous near the substrate where B_z drops by an order of magnitude whereas the electron density is still high (Fig. R8 (b)). A small amount of protons present in the foam region could be accelerated by any vortex fields that might form there. Yet, the acceleration field observed behind the target could be well explained by the TR theory, supported by the excellent agreement between theoretical predictions and simulation results.

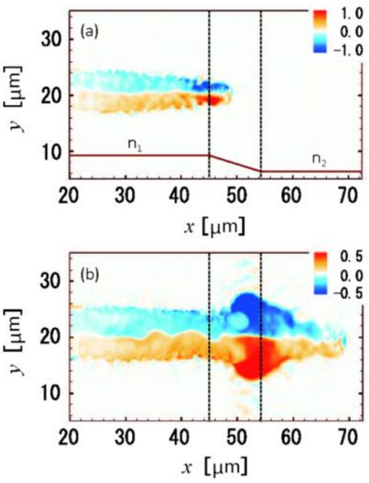

Figure R7. The distributions of magnetic field B_z in gigagauss at (a) t = 220 fs and (b) t = 300 fs. A magnetic dipole vortex is formed at the end of the high density region, and it expands as it moves in the density decreasing region. [Nakamura, et al. Phys. Rev. Lett. 105, 135002 (2010).].

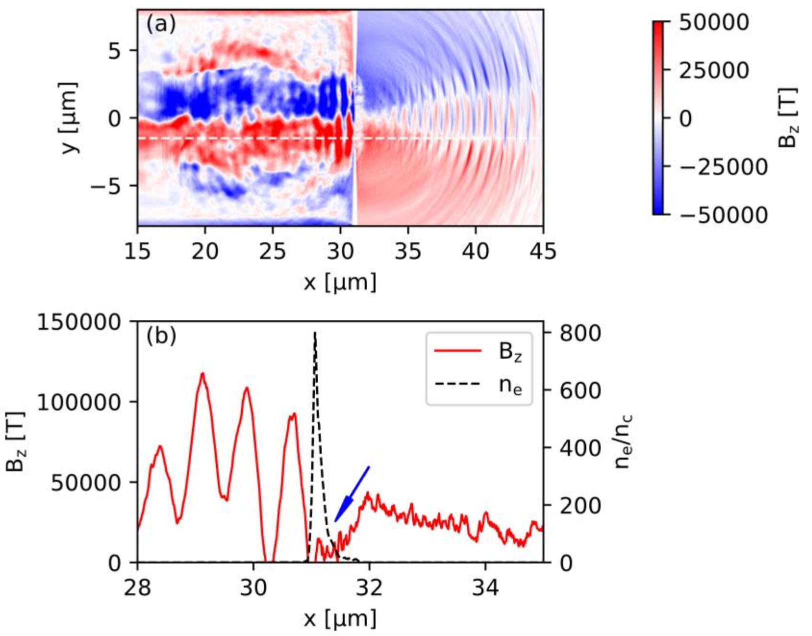

Figure R8. (a) The B_z field of DLA beam traversing the SiN substrate. Laser fields in the foam are filtered out via $B_z + E_y/c$. (b) The B_z field (red-solid) and electron density (black-dashed) on the white-dashed line in (a). The blue arrow indicates the discontinuity/isolation of B_z.

Moreover, the strong magnetic field emphasized by MVA is localized in the NCD plasma, especially in the density decreasing region shown in Fig. R7(a). It does not propagate freely. However, the TRF is a radiated energy, which spreads rapidly from the surface behind the target towards vacuum.

In summary, our theoretical framework accurately decomposes the collective fields into Coulomb and TR components, demonstrating quantitative agreement with 3D PIC simulations across non-relativistic to ultra-relativistic regimes. The analysis conclusively shows that for relativistic, collimated DLA bunches, the TRF dominates proton acceleration while the longitudinal Coulomb field is relativistically suppressed. This insight successfully reconciles previous observations of SPE, revealing them to be predominantly transition radiation effects rather than amplified space-charge fields. Alternative mechanisms such as MVA are effectively precluded due to the isolation of magnetic fields by the SiN substrate. These findings establish TR as a critical, previously underappreciated contribution for proton acceleration.

Changes to the manuscript:

A "Theoretical calculation of TRF" section is added to enhance the theory details. (See lines 186-226)

Clarify on why MVA is not a key consideration in our experiment. (See lines 140-145)

Comment 2: The estimation of TRF strength from THz measurements deserves more detailed justification. The authors present convincing data showing sub-joule THz emission energies, correlated with electron charge, and they translate these into fields of 10^12–10^13 V/m. However, this translation relies on assumptions about source size, emission geometry, and temporal duration. The manuscript would benefit from a more transparent treatment of uncertainties. For example, the error bars in Figure 3(a)(b) could be expanded to explicitly show measurement and calibration uncertainties. A brief sensitivity analysis of how variations in assumed beam diameter or pulse duration affect the estimated field would also add credibility. Importantly, the authors should emphasize that while exact field strengths may have uncertainty, the qualitative scaling with electron charge and foam thickness is robust, which is the key point for their argument.

Response: Indeed, one of the main advances of our work as compared to previous studies is the measurement of TRF in the THz regime. Direct probe of the fields presents significant challenges. Previously proposed mechanisms such as enhanced sheath field or MVA have also faced difficulties in quantifying the field magnitude and distribution. In this work, we performed synchronous diagnose of protons, electrons, and THz radiation. As noted by the Reviewer, we identified the consistent correlations of those signals as a function of the foam thickness, providing a strong support to the TRF enhancement. Furthermore, we attempted to estimate the field strength, thereby providing more straightforward comparisons.

In the near-field region, the electric field strength of TR can be expressed as $E_{acc} \approx \sqrt{\frac{2W_{THz}}{\varepsilon_0 c\pi\tau_{THz} r_{THz}^2}}$, where W_{THz} denotes the radiated energy, τ_{THz} the pulse duration, and r_{THz} the radius of source size [*PNAS* **116**, 3994 (2019)].

The measurement error of THz energy for every shot mainly originates from the peak and average transmittance of the band pass filter (BPF). For example, for a BPF with a

center frequency of 10 THz, the center transmittance is $T_{10\ THz}$ = 0.9 and the average transmittance in FWHM is $0.73T_{10\ THz}$. The BPF transmittance curve usually follows a Gaussian distribution. Errors due to detector calibration and reflections/transmissions from optics/silicon substrates are negligible (consider the frequency within FWHM of the BPF). In the main text of manuscript, we analyzed the results of 30 μm foam, so we further obtained the statistical error of multiple shots. Considering the above factors, the corrected energy is 425.6 ± 67.2 mJ.

The emission geometry of the TRF has been taken into account during the energy calculation. We kindly ask you to refer to the response of comment 2 for Review 2.

The lateral frequency uncertainty in Fig. 3b, originating from the BPF bandwidth, may introduce errors in duration estimation. Following the Reviewer's suggestion, we have refined this analysis. Theoretically, the pulse width should be comparable to that of the escaping DLA electron beam, which simulations indicate to be ~93 fs (~28 μm). After accounting for the BPF bandwidth, the Gaussian fitting range is indicated by the red region in Fig. R9. The experimentally measured duration falls within 83±22 fs, in reasonable agreement with simulation results.

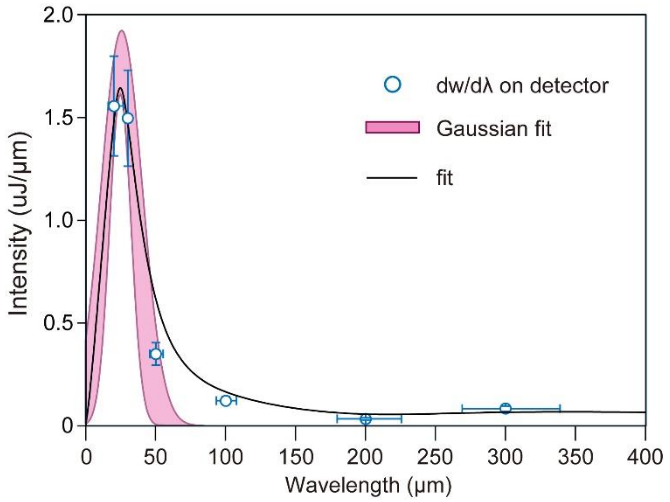

Figure R9. THz wave spectrum. The uncertainty in the duration can be determined by the red region.

Direct measurement of the transition radiation source size is challenging. Therefore, we provide a reasonable estimate based on PIC simulations. As the DLA electron beam exits the foam plasma channel, its transverse size is comparable to the plasma channel diameter,

i.e., about 10 μm at the rear target surface.

A brief sensitivity analysis was conducted. The error transfer function is $\left(\frac{\Delta E_{THz}}{E_0}\right)^2 = \frac{1}{4}\left(\frac{\Delta W_{THz}}{W_0}\right)^2 + \frac{1}{4}\left(\frac{\Delta \tau_{THz}}{\tau_0}\right)^2 + \left(\frac{\Delta r_{THz}}{r_0}\right)^2$. The TRF can be estimated as $E_{THz} = E_0 \pm \Delta E_{THz}$. It is obvious that fluctuations in radius r_{THz} is more influential than other parameters. Thus, TRF is highly sensitive to laser focal spot size and plasma density. Here, consider $r_{THz} = 5\ \mu m$, which is equivalent to the radius of the plasma channel from PIC simulation, W_{THz} = 425.6 ± 67.2 mJ and τ_{THz} = 83±22 fs, then TRF ~ (7.0 ± 2.2) × 10^{12} V/m.

Changes to the manuscript:

We have refined the estimation of the TRF, introduced the expression for E_{acc} and a concise sensitivity analysis. (See lines 157-173)

In the caption of Fig. 3, we highlight the sources of error in the electron and THz data.

Figure 7 and the uncertainty in the duration. (See lines 162)

Comment 3: The discussion of optimal foam thickness could be elaborated. The authors attribute the existence of an optimal thickness (~35 μm) to a trade-off between sufficient interaction length for electron acceleration and the onset of laser filamentation in thicker foams. This is a plausible explanation, but currently the evidence is limited to one simulation of laser channeling. More systematic simulation results could better support the argument. At minimum, the authors should include a clearer physical picture of how laser energy depletion and filamentation reduce electron collimation and, in turn, limit TRF generation. Adding a schematic or summary graph linking foam thickness to expected propagation regime would help readers understand why the optimum occurs where it does. Maybe it is better to show a large range of simulation domain from x=20 to x=40 um for the results in Figure 4.

Response: We thank the Reviewer for this valuable suggestion. In Fig. R10, we have now plotted the spatial distributions of the laser field and electron density at different time when laser propagates in foam. Under the parameters considered in our study, it can be observed that the laser pulse bifurcates after propagating approximately 40 μm, followed by a rapid decay in the laser field intensity, the energy on substrate is lowered. Thereafter, an obvious

degradation in both the energy and collimation of the electron beam is also evident, which, in turn, limits TRF generation. Accordingly, the balance between sufficient interaction length and stable laser propagation is crucial for maximizing the role of electrons in the sheath field and TRF.

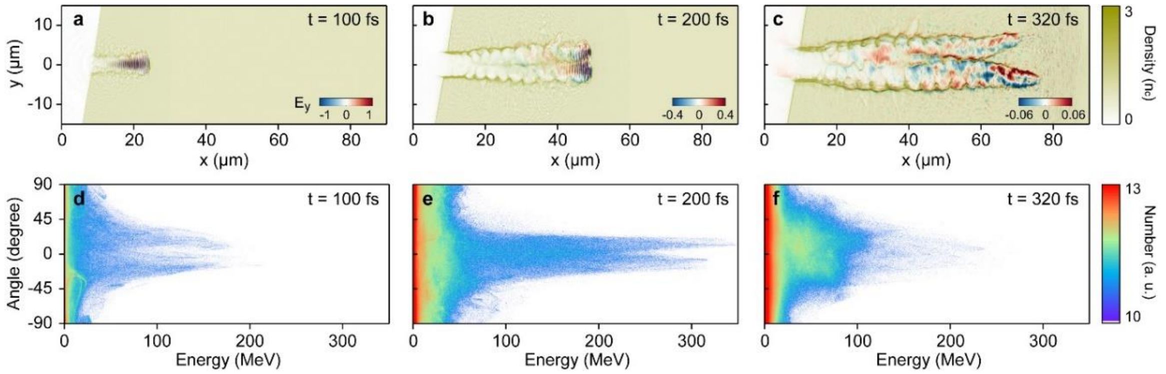

Figure R10. Temporal evolution of the laser and electron beam. **a-c** Distribution of the laser field and electron density in CNF at different times. They are normalized to the laser electric field E_0 and critical density n_c, respectively. **d-f** Angular distribution of electron beams.

Changes to the manuscript:

Figure 6 and its related discussion. (See lines 313-329)

Comment 4: The authors decouple TRF from the sheath field by running reference simulations without foam and subtracting the resulting sheath field from the total field in foam-included runs. This approach is reasonable, but the underlying assumptions should be better communicated. For instance, the linearity of superposition may not be perfect in a nonlinear plasma system. The authors should state explicitly that their method provides an approximate rather than exact separation of fields, and they should argue why it is nevertheless meaningful. Including numerical convergence tests, for example, showing that box size, resolution, or particle number do not qualitatively alter result, would also reassure readers. In addition, the phase-space plots of protons under TRF-enhanced versus sheath-only conditions are compelling; expanding these comparisons and linking them more directly to the experimental plateau spectrum would make the case for TRF much more persuasive.

Response:

We thank the Reviewer for these insightful suggestions. We have compared and

explained theory and simulation results earlier, thereby demonstrating that the TRF dominates when relativistic electrons traverse the rear surface. The theoretical model was first employed under ideal conditions, i.e., considering direct interaction between the electron beam and the target. To make a more realistic comparison, the TRF from simulation must be extracted, which is difficult since TR overlaps with Coulomb fields as discussed in previous response. Therefore, we run a reference simulation with depleted laser pulse interacting with the substrate (without foam) to get an approximation of the effective sheath field and reveal the contribution of the TRF. This approach is employed under some approximations. For example, the TRF could be strong enough to deform the substrate rear. These effects are not included in the reference simulation without foam. When calculating the TRF, the model also neglects the transverse momentum of DLA electrons (as mentioned previously). Still, these effects do not undermine our conclusions. As shown in Figs. 4(d-f), the extracted TRF agrees well with our calibrated theoretical calculation, showing consistent shape and amplitude for the longitudinal component. We have included these approximations in the revised manuscript.

Changes to the manuscript:

The above discussion is added to the Simulation part of Methods section. (See lines 405-416)

Numerical convergence of the 3D-PIC simulations is carried out. We have performed higher-resolution simulation by increasing the resolution from 0.01μm × 0.02μm × 0.02μm to 0.01μm × 0.01μm × 0.01μm to verify that our results are robust. The comparison of fields and final proton spectrum are shown in Fig. R11. The consistency confirms the validity of the chosen resolution.

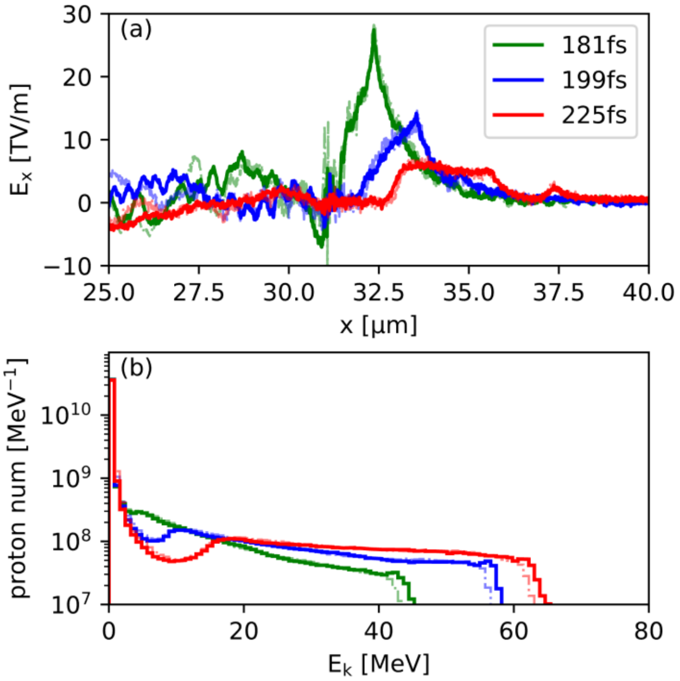

Figure R11. High-resolution results of (a) the on-axis E_x field and (b) the proton energy spectrum at 181 fs (green), 199 fs (blue) and 225 fs (red). The dotted-dashed lines are the high-resolution results.

In the following, we provide a more detailed analysis on how TRF induces spectral plateau. In general, the TNSA-accelerated protons have a Maxwellian spectral profile. This is because in the well-known plasma expansion model of TNSA [P. Mora, Phys. Rev. Lett. 90, 185002 (2003)], the proton front where the most energetic protons sit, co-moves with the peak of the sheath field. Behind the peak is the declining region of the sheath field, where protons of lesser energies are located. As a result, protons of higher energies always witness stronger sheath field, forming a self-similar evolution and hence Maxwellian spectral profile. Conversely, in the light pressure acceleration scenario [Macchi, A., et al. Phys. Rev. Lett. 103, 085003 (2009).], protons of higher energies are located in the weaker region of the acceleration field, eventually leading to overall similar acceleration gradient for most protons and hence quasi-monoenergetic spectrum.

In our case, the presence of TR on top of TNSA induces significant spectral modulation. Although the DLA electron bunches each propagate at velocities close to the speed of light, the TRF is sustained for a duration corresponding to the length of the total electron stream,

comparable to the laser pulse length. Furthermore, the TRF extends to a relatively large scale also determined by the length of the full electron stream. This can be seen clearly in Fig. R12. As the sheath field decays, the dominating TRF formed by the long DLA electron bunch launches from the target rear and gradually sweep through the protons pre-accelerated by sheath field, as shown in Fig. R12 (b) and (c). The field decays as it propagates thus protons closer to the target rear (with less kinetic energies) witness stronger acceleration. Eventually, these protons catch up with the ones of higher energies. As a result, the number of protons in the high energy region grows, leading to flattened spectrum profile. To further illustrate this, we track the per-proton energy and acceleration gradient evolution in Fig. R13. In Fig. R13(a) at 176 fs, purple (near target) protons have relatively low energies; yellow (further from target) protons have higher energy, which still shows characteristic of TNSA. In the following 40 fs, some low-energy protons (purple) get accelerated to mid-high-energies due to the TRF. It is clear in Fig. R13(b) that these protons experience higher acceleration gradient. This shapes the spectrum profile by shifting the low energy protons to the higher energy region, creating the characteristic plateau structure.

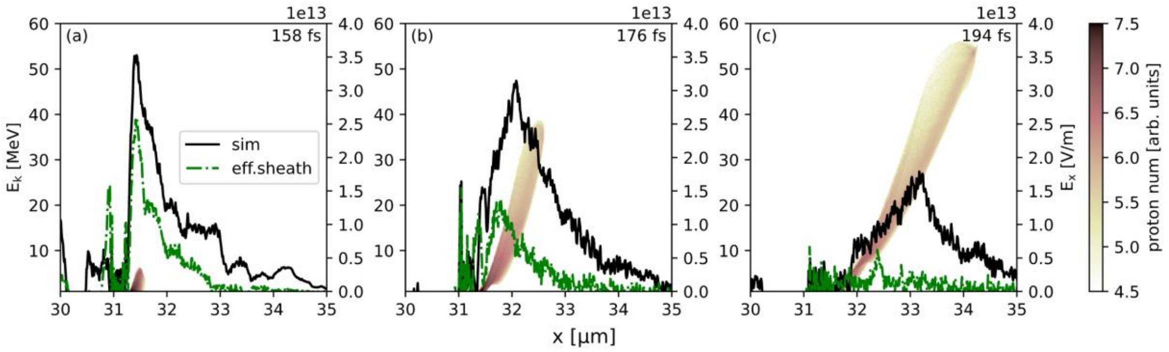

Figure R12. Evolution of the E_x field (black-solid), effective sheath field (green-dash-dotted) and proton in the (x, E_k) space (color map) at (a) 158 fs, (b) 176 fs and (c) 194 fs.

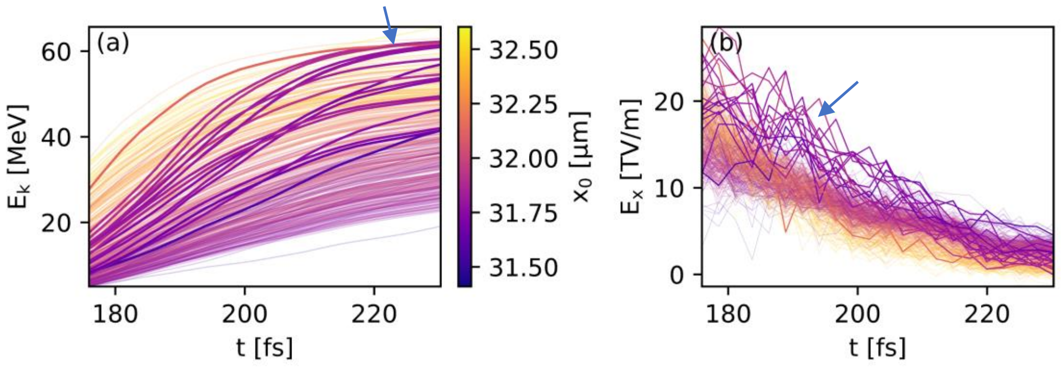

Figure R13. (a) Evolution of proton Ek in the 176-230 fs time range. (b) Evolution of acceleration gradient of protons. Arrows annotate the protons experiencing high acceleration fields.

Changes to the manuscript:

Figs. 5(c-f) and the discussion of the plateau. (See lines 263-287)

Comment 5: Although the manuscript is well written, some passages could be clarified for a broader readership. The equations describing TRF fields (Eqs. 1–2) are mathematically correct but are introduced somewhat abruptly, with little physical explanation. Many readers of Nature Communications may not be familiar with transition radiation theory. The authors could improve accessibility by providing a short intuitive description, for example, explaining that TRF arises when a relativistic electron bunch crosses a plasma vacuum interface, generating coherent radiation because the bunch is much shorter than the radiation wavelength. Similarly, the experimental setup descriptions could be streamlined, focusing on the essential features rather than technical details of filters or detector models, which could be moved to Methods or Supplemetary information.

Response: We thank the Reviewer for this excellent suggestion to improve accessibility. We have added an intuitive physical description of transition radiation fields before introducing Eqs. (1-2) in the manuscript.

Transition radiation arises fundamentally from the requirement that electromagnetic fields must rearrange when a charged particle crosses a boundary between media with different dielectric properties. When a relativistic electron bunch exits the plasma and enters vacuum at the target rear surface, the field configuration cannot instantaneously adjust to the new boundary conditions. This misalignment generates a radiation pulse that propagates

outward with the bunch. The radiation becomes coherent when the electron bunch length is much shorter than the emitted radiation wavelength. In our experiments, the DLA electron bunch has a duration of approximately 93 fs (28 μm), which is indeed notably shorter than the THz wavelengths we observe (20 μm to 3 mm for 0.1-15 THz), satisfying this coherence condition and enabling intense TRF emission.

The revised manuscript now presents a clearer physical picture: as the high-charge DLA electron bunch crosses the plasma-vacuum interface, it generates a coherent TRF that propagates with the bunch, creating an extended acceleration region where the radiation field overlaps with and enhances the existing sheath field. This synergistic combination produces both the observed energy enhancement and the characteristic plateau structure in the proton spectrum.

We have streamlined the experimental setup description in the main text, moving technical details about detectors to the Methods section, as suggested.

Changes to the manuscript:

Introduction about TRF theory has been added. (See lines 186-226)

Some technical details about detectors have been moved to Methods section. (See lines 346-361)

Comment 6: For applications, the manuscript mentions medical therapy and compact accelerators as motivations. While such aspirations are understandable, the current results remain below the energy thresholds (~150–200 MeV) required for clinical turmor therapy, and the plateau spectrum may not be optimal for narrow-bandwidth applications. The authors should moderate their claims, presenting applications as long term motivations rather than immediate prospects. Instead, they could emphasize the conceptual importance of discovering a new field contribution to proton acceleration, which may inspire further optimization. Such framing will strengthen credibility and avoid overstatement.

Response: Our work indeed aims to highlight a new understanding of the proton acceleration fields. As rightly noted by the Reviewer, the application of laser-driven proton beams in tumor therapy remains a long-term vision, as currently achieved cutoff energies still fall short of clinical requirements.

In the opening paragraph, we outline the research background of laser-driven proton acceleration, including its potential applications and the cutoff energies attained under mainstream acceleration mechanisms. The subsequent paragraph emphasizes the importance of clarifying the underlying acceleration mechanisms to further enhance proton energies. In the following section, we highlight the discovery of new physical mechanisms in our study. These findings provide a meaningful extension to previous work and constitute the core contribution of this paper.

We balance the description and update the abstract and introduction parts of manuscript accordingly.

Changes to the manuscript:

Abstract: Laser-driven protons with ultrafast temporal properties attract great interest in fields ranging from flash radiation oncology to compact accelerators. High-effective energy coupling of protons from laser-induced accelerating fields is complex, hybrid acceleration mechanisms that combine multiple field contributions proves critical for optimizing proton energy. (See lines 16-19)

Introduction: Laser-driven proton sources have been extensively studied because of their potential applications in probing high-energy density states[1], radiation oncology[2], fast ignition fusion[3], and laser nuclear physics[4]. (See lines 36-37)

Comment 7: Minors.

Response: We thank the Reviewer for the careful review and our responses to these minor points are as follows.

1) Line 64: The work Ref. [31] shows that CNF can achieve a long acceleration time without sacrificing the strength of the acceleration field by utilizing composite targets. The strength of the accelerating electric field is not enhanced in the scenarios of Ref. [31].

We have carefully reviewed the Ref [31] and revised the manuscript.

Changes to the manuscript:

Experiments have shown that CNF can achieve a ~2 times increase in proton energy and enhance heavy-ion acceleration through improving the strength of charge-separation field or the acceleration time[18, 19, 31]. (See lines 64-66)

2) Line 101: The proton energy spectrum in Figure 2(b) and 1(g) shows that the cutoff energy can approach 90 MeV, which is measured by using thomson parabola spectrometer (TPS). What about the experimental raw data of cutoff proton energy measured by the stack detector, such as CR-39 plates and radiochromic films (RCFs)?

We primarily employed a TPS to determine the proton cutoff energy, while RCF stacks were used in limited number of shots to characterize the proton spatial distribution. It should be noted that the 90-MeV proton data were obtained solely from TPS measurements. We also used the same TPS to measure protons in our previous work such as the Ref. High Power Laser Sci. Eng. 10(10): e26 (2022)).

3) Line 241: It might be helpful for understanding the acceleration mechanism by adding a subfigure to show the distribution of protons in the (x, px) phase space?

We thank the Reviewer for the suggestion. Fig. 5 is updated.

Changes to the manuscript:

The evolution in (x, px) space is added to Figs. 5(c-d) of the revised manuscript.

4) Line 283: Is the pulse duration of ~30 fs measured at the FWHM of the laser intensity? The abbreviation 'FWHM' is not defined in the manuscript.

Yes, it is.

Changes to the manuscript:

We have defined the abbreviation ‘FWHM’. (See line 87)

5) Line 294: There should be some compatible problems. The sentence "Error! Reference source not found.a" does not make sense.

Revised.

6) Line 276: It is better to set the white-green colorbar to be transparent for the part with 5 MeV to enhance the presentation.

Fig.6 is updated to 2D form.

We again thank you for the thoughtful comments, which helped us significantly improve the quality of our manuscript. We hope that we have addressed all your concerns.

Reviewer #2

General comment: The manuscript by Qin et al reports proton cut-off energies up to ~90 MeV by irradiating PW Ti:Sa laser pulses onto near-critical density (NCD) foams layered on 100 nm SiN substrates. Varying foam thickness, they identify an optimum regime that yields a pronounced high-energy plateau in the proton spectra. The central interpretation is that, at this optimum, a high-charge relativistic electron bunch generates a coherent transition-radiation field (TRF) at the substrate rearside which overlaps and widens the rear-side accelerating region, thereby boosting proton energies and generating the high-energy plateau in the proton spectrum. For this optimum foam thickness, they report rear-emitted THz energies up to ~0.6 J across 0.1–15 THz and electron bunch charges up to ~26 nC. PIC simulations are used to argue that the TRF amplitudes can reach 10^{12} V/m, thereby significantly contributing to the proton acceleration.

The experimental dataset is interesting and showcases the current performance of their PW laser system with NCD foam-targets. However, in the context of today's state of the art, the reported ~90 MeV from 56 J is not, by itself, a high-impact outlier: recent experiments have reached ~150 MeV with ~22 J via cascaded transparency regimes [Ziegler et al., Nature Physics (2024] and >110 MeV using conduction-restricted nanometer foils [Shou et al., Nat. Commun. (2025)]. NCD foams and double-layer targets are already established routes for boosting electron and ion energies, with multiple experiments attributing the gains chiefly to superponderomotive electrons that strengthen the sheath fields [Bin et al., PRL (2018); Ma et al. PRL (2019)]. Intense X-ray emission from foams and the use of THz diagnostics to probe ultrafast laser–target dynamics are likewise published (e.g. [Shou et al., Opt. Lett. (2021); Tavana et al., Frontiers in Physics (2023); Liao et al., PRL (2024)]). What the authors claim to be new in their manuscript is the interpretation that a coherent TRF contribution enhances the proton acceleration. The paper's impact therefore hinges on convincingly establishing that TRF contribution beyond reasonable alternatives.

Response: We thank the Reviewer for the careful review of our work, and positive assessment of the highlights distinguish from recent reports.

We employed a laser intensity that lies between the intensities of [Ziegler et al., Nat. Phys. (2024)] and [Shou et al., Nat. Comm. (2025)]. The laser system and target configuration are also quite different from the above works. The strength of our work, as acknowledged by the Reviewer, is revealing the contribution of TRF in laser-proton acceleration. We thus focus on the new acceleration component, which suggests an optimization strategy that was previously unexplored.

The use of NCD foams and double-layer targets in laser-proton acceleration have been demonstrated [Bin et al., PRL (2018); Ma et al. PRL (2019)]. In TNSA, the so-called sheath field comes from the expansion of hot plasma which produces a charge-separation field (CSF) between the ion front and the electron cloud [Mora, Phys. Rev. Lett. 90, 185002 (2003)]. The introduction of NCD foams provides superponderomotive electrons (SPEs) from direct laser acceleration (DLA), which are in general relativistic and quite directional. Previous work attributed the increase of proton cut-off energies to the "enhanced sheath field" by these DLA electrons. Yet it was unclear how the SPEs are coupled into the field. We notice that such highly directional relativistic DLA electrons contribute to the acceleration field in several ways. It cannot simply be regarded as pure CSF since they are drastically different from expanding thermalized electron clouds. They transverse the plasma boundary, escape from the plasma bulk and propagate into vacuum, i.e., SPEs are not dragged back to plasma and thermalized like most ponderomotive electrons in TNSA [Sentoku, Phys. Plasma 10 2009 (2003)]. To find out the how sheath field is enhanced by these electrons, we start from first-of-principal calculation by looking at the Lienard-Wiechert potential associated with these electrons transmitting the plasma boundary. These fields are naturally decomposed into the Coulomb field and the Radiation field, corresponding to the CSF and transition radiation field (TRF) components. Immediately, we see that when the electrons are sub-relativistic, the CSF dominates. However, for most DLA electrons that are well above the relativistic threshold, the TRF surpasses the CSF and becomes the dominating contribution. Presence of the TRF increases the proton energy on top of the enhanced CSF (driven by low-energy electrons) and further induces plateau energy spectra. To distinguish the contribution of TRF, we name it TNSA-TRFA.

The induced energy enhancement and plateau spectra are clearly seen in our

experiments. We went one step further, by measuring electron beams and TRF in the THz regime, in additional to the proton signals. This is, to the best of our knowledge, the first systematic measurement on the correlation between the proton acceleration, the DLA electrons and the THz radiation. A most important result is that we identified consistent dependence of the proton cut-off energy, the TRF energy and the electron beam charge as a function of the foam thickness. We estimated the field strength of the TRF based on the measured THz pulse energy, the derived pulse duration and the approximated source size. These estimations, although not perfect, show the near-field TRF reaching 10^{12}-10^{13} V/m, in reasonable agreement with the energy gain and the full 3D PIC simulations.

Here, we would also like to mention that magnetic vortex acceleration (MVA) is relatively weak under our experimental conditions. First, according to the idea of MVA - like the one proposed in Ref. [Phys. Rev. Lett. 98, 049504 (2007)], the time-varying magnetic field, generated by currents during laser propagation through low-density plasma, typically contributes significantly less to the acceleration field than the sheath field does. Moreover, typical MVA, as described in Ref. [Phys. Rev. Lett. 105, 135002 (2010)], usually requires several-micron-thick (10 μm), low-density (~0.3 n_c) plasma behind the NCD target to generate a strong magnetic field. In our experiments, the NCD foam is attached to a 500-n_c solid-density substrate, which effectively prevents the laser penetration (see the response of Comment 1) and isolates the strong magnetic field in the plasma channel from the acceleration region (please see also Figs. R7-R8 our response to comment 1 of Reviewer 1). These distinctly different plasma conditions are unfavorable for MVA. Second, MVA proton beams are highly collimated. As shown in Fig. R14, the full angle is 1~3° for high energy protons when laser power is high. However, our proton diagnostic (TPS) is positioned at 5 degrees deviating laser direction, making it less likely to capture the highly directional and energetic protons. Third, from the perspective of magnetic field characteristics, MVA features localized magnetic fields. In our study, the strong magnetic fields are components of the TRF, thus propagating at the light speed towards vacuum.

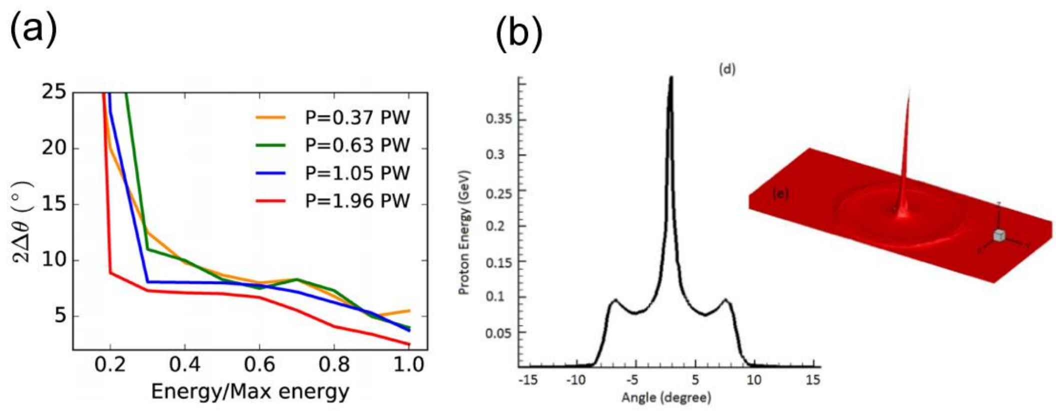

Figure R14. (a) Typical divergence angle of MVA protons with different normalized energy (Fig.5 in [Phys. Plasmas 26, 103108 (2019)]). (b) Proton density distribution in MVA ([Sci. Rep. 8, 2191 (2018)]).

As the Reviewer suggests, the primary significance of our work lies in serving as a new technical piece that helps decipher the components of complex acceleration fields. We believe the above systematic analysis and experimental effort go beyond previous studies and sort out the mystery of how exactly the sheath field is enhanced by DLA electrons. This perspective receives limited attention in earlier studies. With the increasing availability of femtosecond petawatt-class lasers and the growing use of near-critical-density targets, near-field TRF should play an increasingly important role, especially when protons approach the speed of light to be better synchronized with the TRF.

We have implemented the above discussion and more experimental details (as required by the Reviewer in the following comments) in the revised manuscript to strengthen the findings we made.

Comment 1: Shot-to-shot stability and causality. THz, electron, and proton data were not recorded on the same shots and, critically, were taken at different laser energies (≈56 J for ions/electrons vs ≈68 J for THz—>20% higher), so the reported correlations are indirect. Please also clarify whether transmitted 800 nm light was detected behind the 100 nm substrate; at the stated peak intensities partial transparency is plausible and would affect the rear conditions. In addition, the total number of shots per setting and precise error-bar definitions are missing in Figs. 2 – 3; mean values can be misleading when the total number of shots is small.

Response: We thank the Reviewer for this precise comment. We now address your concerns in the following.

a) THz energy.

In Figs. 2–3, the data for THz radiation, electrons, and protons are correlated through the same target parameters. In the revised manuscript, we have associated the THz data obtained under the 68 J laser condition with the 56 J laser condition, allowing direct comparison among the three datasets. At 68 J, the laser-to-THz energy conversion efficiency is measured to be (0.76 ± 0.12) % for 30 μ m foam. Assuming similar conversion efficiency, the THz energy under the 56 J laser condition is estimated to be 425.6 ± 67.2 mJ. Accordingly, the near-target intensity and acceleration gradient induced by THz radiation have been recalculated based on the scaled THz data.

b) Laser penetration problem.

Plasma mirrors were used to improve the laser temporal contrast in experiment. No significant laser penetration was observed in our experiments. Figure R15 shows the images of the Aluminum foil placed before the stack. For SiN substrates of 30 nm and 50 nm thickness, notable laser ablation on the Al foil is observed. In contrast, the 100 nm SiN substrate alone and together with the foam target exhibits no signs of laser ablation. This is because the SiN membrane is of much higher electron density (~500 n_c), which is 2–3 times higher than that of a CH target. The latter would be broken through by the multi-PW laser based on our previous experiments (not shown here).

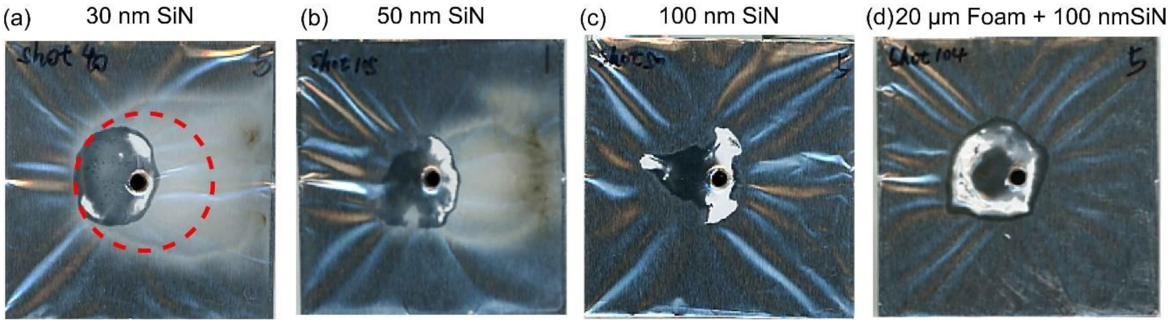

Figure R15. Aluminum foil images after laser or particle beam irradiation. The red circle in (a) marks the laser contour in case of free propagation.

c) Number of shots and error bars.

We appreciate the careful review. In the revised version, we have provided more detailed descriptions regarding shot numbers and error bars. In Figs 2 and 3, the electron and proton data points with error bars are derived from 2 – 3 shots. Both 2D and 3D PIC simulations further confirm proton energy variations with target thickness that are consistent with the experimental results. For the THz data, shot-to-shot fluctuations are relatively small, and the larger uncertainty mainly originates from diagnostic instrumentation errors, which have also been explained in the corresponding captions.

Changes to the manuscript:

THz energy analysis under 56-J laser condition. (See lines 157-159)

Clarify of the shoots in caption of Fig. 2.

Comment 2: Quality of experimental data. The electron IP stack at 5 cm is highly vulnerable to contamination by X-/γ-rays from the foam/target and bremsstrahlung from ions/electrons in upstream absorbers. Electron specificity has not been demonstrated. Established controls—photon/electron discrimination using additional magnetic deflection and shielding, plus Monte-Carlo dose estimates for the exact geometry—are needed, with the resulting systematics propagated into any correlations. For the THz diagnostics, a complete calibration is required: full optical transfer function and geometric acceptance with uncertainties, and an independent energy/time cross-check. The pulse-duration estimate derived from channelized spectra is heuristic and should be justified or replaced by a defensible time-domain retrieval within the stated bandwidth limits.

Response: In the following, we perform thorough analyze of the probed electron and THz signal, together with the estimation of the TRF pulse duration.

a) Assessment of the impact of X/gamma ray on electron analysis.

Employing magnetic fields to separate photons and electrons indeed constitutes an effective approach for accurately measuring electron energy spectra or counts. However, due to the large divergence angle of the electron beam (with a full angle approaching 50°), measuring the entire electron flux imposes stringent requirements on the magnetic spectrometer. Using a metallic stack to measure electrons is also a widely adopted general method. We have evaluated the influence of photons on electron diagnostics and have made

efforts to minimize their impact during data processing.

First, the X/gamma rays generated by the foam target can be regarded as background noise, which is attenuated by the stack through absorption. Depending on the specific stack design, adjacent image plates (IPs) are separated by iron sheets with thicknesses ranging from 1.8 to 3.2 mm, capable of blocking photons with energies of 53–67 keV and electrons with energies of 2.1–3.2 MeV. Consequently, such thin iron layers provide significantly less attenuation for photons than for electrons, resulting in comparable photon intensities on neighboring IPs. The PSL signal on the first two IPs contains both electron and photon components, $PSL_n = P_{n,\,ele} + P_{n,\,pho}$, $PSL_{n+1} = P_{n+1,\,ele} + P_{n+1,\,pho}$. Consider $P_{n,\,pho} \approx P_{n+1,\,pho}$, then $dN_n = (PSL_n - PSL_{n+1})/R_{ele} \approx (P_{n,\,ele} - P_{n+1,\,ele})/R_{ele}$, where $P_{n,\,ele/pho}$, R_{ele} and dN_n are the PSL value of electron/photon, IP responsiveness of electrons and difference in the number of adjacent IPs. So when calculating dN_n, the noise impact is minimized as much as possible. In fact, the Bremsstrahlung of ions can be neglected due to their large mass and low energy.

Second, when the electron beam bombards the stack, it can generate X/gamma rays, making the stack a source of such radiation. Following the Reviewer's suggestion, we have included Geant4 simulations to quantify the photon influence. As shown in Fig. R16(a), the PSL signals produced by transmitted electrons and X/gamma rays on the IPs are simulated using an electron beam with a temperature of 15 MeV and a cutoff energy of 60 MeV incident on the stack. Figure R16(b) presents the IP-SR response curves for both types of particles, indicating that the IP response to X-rays is significantly lower than that to electrons with energy > 0.1MeV. Photons with energy greater than 0.1 MeV can avoid absorption by the filter itself. As can be seen, the PSL value induced by photons on each IP is relatively small and decays slowly in intensity. Similarly, in solving for dN_n, the interference from photon signals is significantly suppressed. The proportion of photon signals on dN_n can be expressed as $Pr_n = (P_{n,\,pho} - P_{n+1,\,pho})/(PSL_n - PSL_{n+1})$. Considering the first two IP layers of 35 μm foam case, $Pr_1 = 1.5\%$. As the electron beam decays, the value for Pr_7 increases to 5%. Thus, the signal interference induced by X/gamma rays is approximately one order of magnitude lower than that from electrons.

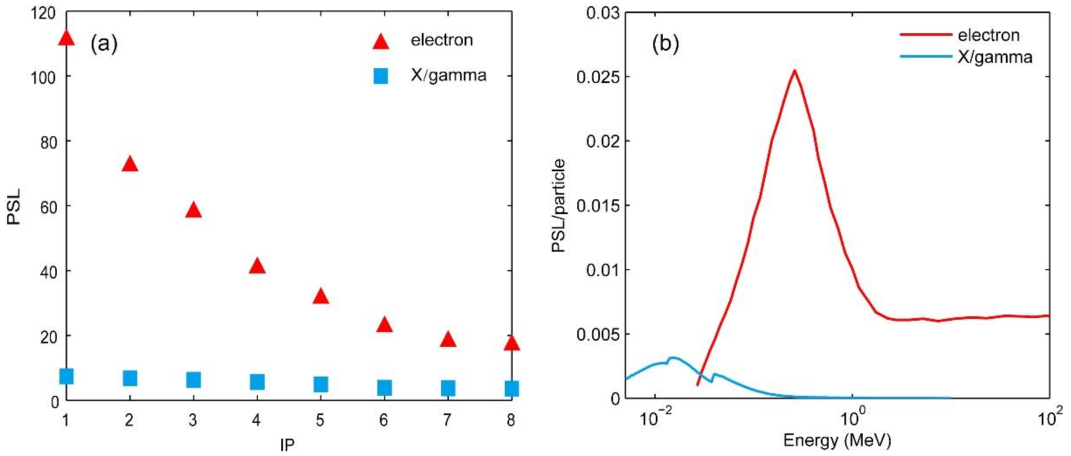

Figure R16. (a) The PSL value induced by electron and X/gamma ray per IP. Obtained according to Geant4 simulations. (b) Response of the image plate to electrons and photons. [Rev. Sci. Instrum. 86, 113304 (2015), Rev. Sci. Instrum. 84, 103510 (2013)] Red denotes electrons, and light blue denotes X/gamma ray.

In addition, considering the weak attenuation of photons by the stack, we take the PSL value at the edge of the reference IP furthest downstream as the overall background noise, and subtract it when solving for dN_n, thereby further reducing interference. Overall, the influence of photon signals on electron spectrum analysis is very small. We have further clarified this point in the Methods section of the revised manuscript.

b) THz analysis.

Regarding the terahertz measurements noted by the Reviewers, we already provide a complete description in the Methods section in the first manuscript. Here, we further clarify the numerical details. The optical transfer function/peak transmittance across the full optical link is T = [0.186, 0.056, 0.050, 0.046, 0.097, 0.095, 0.080, 0.068, 0.024]. Considering the Gaussian-like transmittance function of the band pass filter (BPF), the average transmittance within the unit bandwidth is 0.73T, which contributes dominantly to the overall energy uncertainty. The detector's responsivity R_{det} = [2.6, 2.6, 3.6, 2.2, 2.7, 4.4, 4.3, 4.4, 3], which was obtained directly via independent energy calibration using THz radiation.

Following the typical estimation approaches described in Ref. [*Proc. Natl. Acad. Sci.* **116**, 3994-3999 (2019)], we account for geometric distribution effects when calculating the energy proportion received by the detector. For an collimated electron beam passing through an ideal conductor–vacuum interface, the energy distribution per unit solid angle and unit

bandwidth can be given by $\frac{d^2W}{d\Omega d\omega} \propto \left|\sin\theta \int dug(u)F(\omega,\theta,u)\ \frac{u(1+u^2)^{1/2}}{1+(u\sin\theta)^2}\right|^2$, where g(u) is the momentum distribution function, u is the normalized momentum and $F(\omega,\theta,u)$ is the spatial form factor [Phys. Rev. E 69, 016501 (2004)]. Figure R17(a) shows the calculated intensity distribution of the THz. It takes the form of a ring-shaped optical structure, whose geometry is consistent with the previously experimentally observed transition radiation [Proc. Natl. Acad. Sci. 116, 3994-3999 (2019), PRL 116, 205003 (2016)]. The detector primarily collects THz radiation within a solid angle of 4×10^{-4} sr at a position of 20°. The intensity distribution simulated by PIC is shown in Fig. R17(b), which exhibits typical circular structure characteristics of transition radiation. The strength is expressed as $E_{intensity} = \sqrt{E_x^2 + E_y^2 + E_z^2}$.

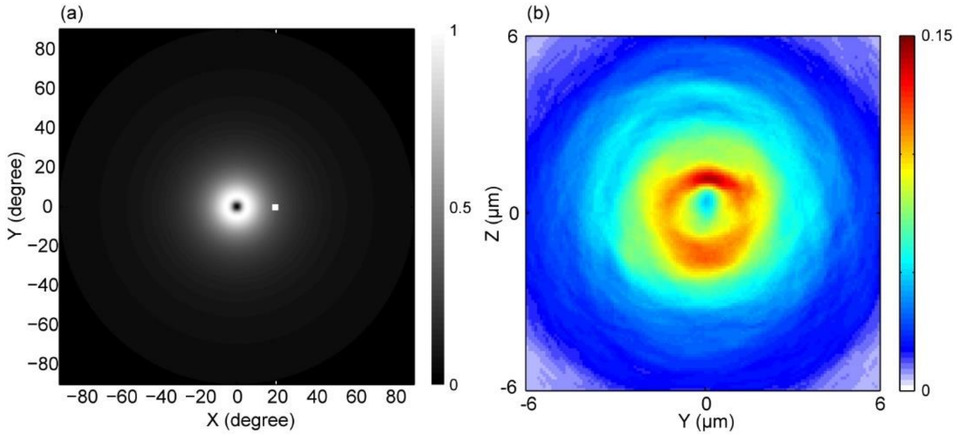

Figure R17. (a) Theoretically evaluated normalized angular distribution of THz radiation driven by a electron beam with T_e = 15MeV. The white square indicates the position of the detector, not the actual size. (b) Cross sectional distribution of electric field strength near the SiN substrate referring to PIC simulations. It is normalized to laser electric field.

c) The pulse-duration estimate form time spectrum

It is a sound suggestion to convert frequency signals to the time-domain via inverse Fourier transform. However, due to our measurement methodology, it is rather challenging to obtain accurate time-domain signals. The multi-channel THz spectrometer introduces severe non-uniform sampling, particularly in the high-frequency region where the scarcity of data points leads to insufficient temporal resolution and excessively wide frequency intervals. This unknown distribution can easily lead to errors during interpolation fitting. For the wave spectrum, Gaussian fitting can be directly performed according to Ref. [Nat.

Phys. 7, 219-222 (2011)], so we choose this method.

Figure R18 presents the analysis for the high-frequency signal. The red curve represents the interpolated spectrum, and its inverse Fourier transform yields a pulse with a FWHM of ~35 fs. The duration of the high-energy DLA electron bunch is comparable to that of the laser pulse. This estimate aligns in order of magnitude with the pulse width (83 fs) derived from the wave spectrum in our manuscript.

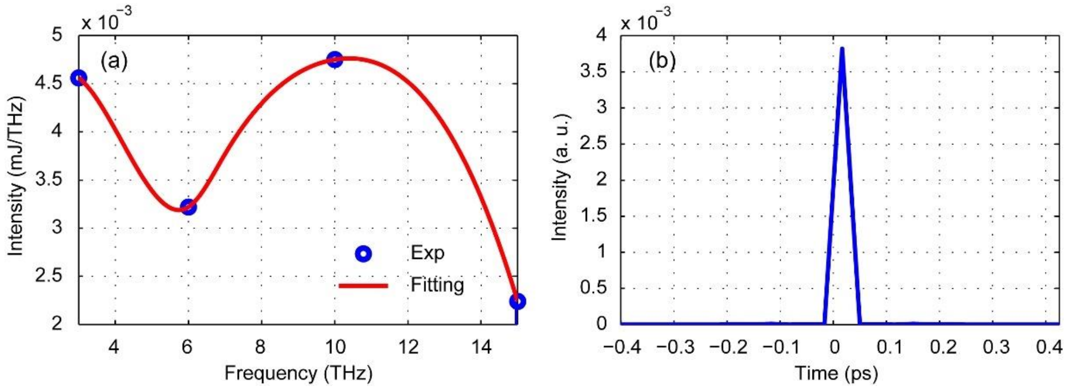

Figure R18. (a) THz spectrum in the high-frequency region, the red curve represents the interpolated spectrum. (b) Time-domain signal obtained after inverse Fourier transform.

Changes to the manuscript:

Some technical details clarify about the IP stack in Methods section. (See lines 346-361)

Figure 7 and the pulse-duration estimate and its error. (See line 162)

The insert in Fig.4 (c) and the related description. (See lines 236-237)

Comment 3: Mechanism ambiguity. There is substantial ambiguity with respect to established explanations—most notably superponderomotive-electron (SPE) enhanced sheaths in double-layer/NCD targets [Bin et al., PRL (2018); Ma et al. PRL (2019)] and magnetic-vortex acceleration (MVA) in near-critical channels. These prior experiments with closely related target concepts described the enhanced acceleration performance to these electrons that amplify the rear sheath; MVA and surface-wave currents can also contribute when long NCD channels and strong azimuthal fields form. In the present manuscript, a systematic classification and delimitation from these mechanisms is missing. The data do not yet show which part of the observed enhancement and plateau uniquely requires a TRF

contribution—versus being explainable by an SPE-dominated sheath or by MVA inside the foam. I do appreciate the general simulation strategy—running a full foam case and a separate "effective sheath" scenario and then comparing to an analytic TRF model—but, as presented, the decomposition is not yet convincing. In particular, the Coulomb (space-charge) fields of the high-charge electron bunches can contribute substantially and are not cleanly separated from the purported radiative TR component. Further discriminating signatures might be available in the authors hypothesis but are not provided. Without discussing and discriminating from alternative interpretations and mechanisms the entire novelty about the TRF contribution remains ambiguous.

Response: We thank the Reviewer for raising this important point about mechanism distinction.

The Reviewer correctly identifies that distinguishing TRF from Coulomb CSF is critical for establishing the novelty of our mechanism. As mentioned in the response to your general comment, although improved proton acceleration was attributed to "enhanced sheaths" via superponderomotive-electron (SPEs) from NCD foams [Bin et al., PRL (2018); Ma et al. PRL (2019)], it is not clear what exactly constitutes to such enhancement. We thus perform systematic analysis based on the first-of-principal calculation from the Lienard-Wiechert potential induced by SPEs. The full discussion is included in the response to comment 1 of Reviewer 1, which we kindly ask you to refer to.

Here we summarize the main results to avoid redundancy. Through quantitative field decomposition framework, we explicitly separate these contributions, demonstrating that TRF dominates under our experimental conditions.

1. Separation of Coulomb and Radiation Fields

The fields associated with electron transversing a conductive boundary (here the plasma-vacuum interface) can be analytically decomposed into Coulomb and radiation components following the Carron model [Carron, PIER 28, 147-183 (2000)]

$$E_x = \frac{Q}{\gamma^2}\left(\frac{1}{S_-}+\frac{1}{S_+}\right)-\frac{2Q}{R},\ 0 < T < \tau_{beam} \quad (1)$$

$$E_x = \frac{Q}{\gamma^2}\left(\frac{1}{S_-}-\frac{1}{S_{l-}}+\frac{1}{S_+}-\frac{1}{S_{l+}}\right),\ T > \tau_{beam}. \quad (2)$$

The $-2Q/R$ term is the TRF (decays as $1/R$, independent of γ), while the $1/\gamma^2$

terms represent the longitudinal Coulomb field (relativistically suppressed). They co-propagate with the electron beam in the relativistic case.

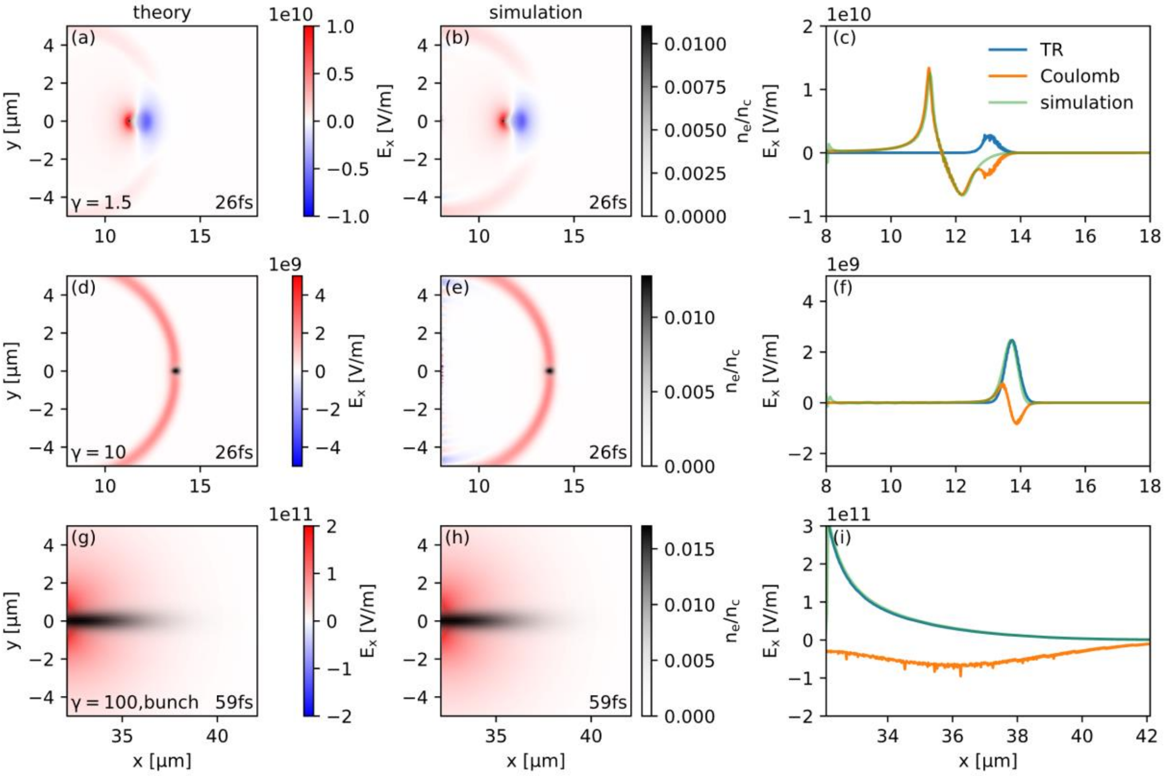

Figure R19. Evolution of transition radiation field for (a-c) non-relativisitc case, (d-f) relativistic case and (g-i) ultra-relativistic bunch. (left) Theoretical TR field. (middle) Simulation results. (right) On-axis acceleration field of the calculated radiation term (TR, blue), calculated Coulomb term (orange) and the simulation result (green). The Coulomb term in (i) is multiplied by 10000 for visibility.

This analytical decomposition is validated against 3D PIC simulations across regimes:

Non-relativistic case ($\gamma = 1.5$): For non-relativistic electrons, there is no suppression of the Coulomb field. Thus, Coulomb field dominates over the TRF (Figs. R19 (a-c)). In this case, the main contribution to the "enhanced sheath" comes from the charge-separation Coulomb component.

Relativistic case ($\gamma = 10$): For relativistic electrons, the longitudinal Coulomb field is suppressed by $1/\gamma^2$, TRF becomes more and more important, and gradually dominates over the Coulomb field. Here both the TRF and the suppressed Coulomb field co-propagate with the bunch (Figs. R19 (d-f)).

Ultra-relativistic bunch ($\gamma = 100$): For highly relativistic electrons, Coulomb contribution is negligible. TRF sustains acceleration until the entire bunch vacates the target (Figs. R19 (g-i)).

For SPEs from DLA in NCD foams, the electron bunch is of broad spectrum and spatially modulated. We thus track the electrons from 3D PIC simulations and obtain the fields following Eq. (1) and (2). The field distribution agrees well with direct simulations (Fig. R5), showing that the TRF is much larger than the Coulomb field. This is because the SPEs are mostly relativistic such that the longitudinal Coulomb field is suppressed.

2. Interpretation of the "Enhanced Sheath" by SPEs

In previous work, the "superponderomotive-electron enhanced sheath" was identified [Bin et al., PRL (2018)]. Yet, it was not clarified how such field is enhanced. We revisited their set-up via both 3D simulations and our analytical model.

The results can be found in Fig. R6. It is clearly seen that the "enhanced sheath" is predominantly induced by TRF, rather than the amplified Coulomb field. Here the DLA electrons are characterized with cutoff energy of around 50 MeV electrons and temperature around 8 MeV, inducing TRF an order of magnitude higher than Coulomb field. This quantitative physical explanation reveals that the enhancement mainly stems from TRF by relativistic electrons, thus solving the mystery in previous works.

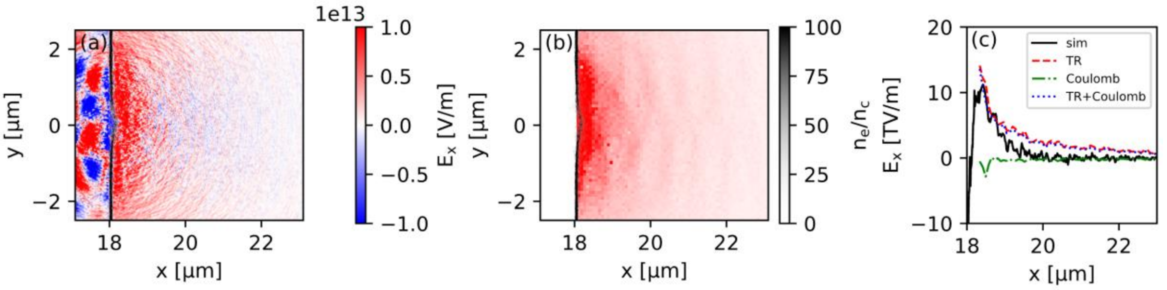

Figure R6. (a) Simulated longitudinal electric field E_x (red-blue) and electron density n_e (black-white) from 3D reconstruction of the configuration in Ref. [Bin et al., Phys. Rev. Lett. 120, 074801 (2018)]. (b) Theoretical calculation using Eqs. (1-2). (c) On-axis field comparison showing the simulation result (black-solid), theoretical radiation term (TR, red-dashed), Coulomb term (green-dotted-dashed), and their superposition (blue-dotted).

3. MVA suppressed by Substrate Shielding

MVA requires laser penetration, channel formation, and expansion of azimuthal magnetic fields in an extended NCD plasma [Nakamura et al., PRL 105, 135002 (2010)]. In our experiment, we employed SiN substrates of electron density 500 n_c and thickness 0.1 μm. We observed no laser penetration through either single SiN foils or SiN with NCD foams (Fig. R15). While a channel is formed in the NCD foam, the magnetic fields are significantly blocked by the SiN substrate, hindering the formation of efficient MVA (Fig. R8). Thus, the set-up is rather unfavorable for MVA. The sustained longitudinal field behind the target in simulations matches well with the TRF theory rather than the MVA model. Furthermore, Our proton diagnostic (TPS) is positioned at 5 degrees deviating laser direction, making it less likely to capture the highly directional and energetic protons.

Changes to the manuscript:

A "Theoretical calculation of TRF" section is added to enhance the theory details. (See lines 1846-226)

Description of SPE-enhanced sheath fields in introduction section and the difference in our work. (See lines 57-58 and 64-68)

Figs. 5(e-f) and discussion on the formation of plateau. (See lines 263-287)

Clarify on why MVA is not a key consideration in our experiment. (See lines 140-145)

Comment 4: Until these issues are addressed, I cannot recommend publication in its present form. The paper may ultimately be stronger if it does not hinge on proving a single "new" mechanism. Reframing the study as a careful, quantitative exploration of how SPE-enhanced sheaths, transition-radiation fields, and possiblef MVA/surface currents interplay in the NCD-foam regime—supported by targeted discriminators—could make a valuable contribution even without asserting one dominant mechanism.

Response:

The use of NCD foam targets can leads to an enhancement of the charge-separation field compared to planar targets. We have cited extensive related work in the Introduction, where such effects have been attributed by previous works to SPE-enhanced sheath fields.

This is usually understood as a charge separation field, and we do not dispute that.

However, for the high-energy DLA electrons, their longitudinal Coulomb field is significantly suppressed due to relativistic effects. We have identified that the major contribution on enhancing proton acceleration lies in generating strong TRF, which represents a novel understanding of the energy coupling between the DLA electron (or SPE) and the accelerating field, and constitutes a key innovation of this work. This also represents a more concrete understanding of SPE/DLA-enhanced proton acceleration.

As we replied earlier, our target settings and diagnostic arrangements are not suitable for typical MVA related discussions.

To more clearly delineate the distinctions among these three mechanisms (charge-separation field/sheath acceleration, TRFA, MVA), and to highlight the unique aspects of our work, we provide further clarification in revised manuscript.

We again appreciate you for the thoughtful and insightful comments, which really helped us in the revision of our manuscript. We hope that we have addressed all your concerns.

Responses to Reviewers' comments

Dear Reviewers,

We truly appreciate the time and thought you have invested in evaluating our work, in both this and the previous round of review. Below, we address the points you have raised and provide a detailed account of the changes made to the manuscript.

Reviewer #1

Comment: I have carefully read the revised manuscript together with the authors' detailed point-by-point responses to the referees' comments. I appreciate the considerable effort the authors have made to address the concerns raised in the first round of review. In my opinion, the responses are thorough and constructive, and the revised manuscript has been clearly improved in terms of clarity, physical interpretation, and supporting analysis.

As said in my previous report, this work represents a potentially strong and important contribution to the field of ion acceleration driven by relativistic laser–plasma interactions. The experimental observations are compelling, and the combination of experimental measurements with three-dimensional PIC simulations provides a solid and comprehensive basis for the conclusions. In particular, the proposed transition radiation field (TRF) assisted acceleration mechanism offers a novel and conceptually interesting explanation for the enhanced longitudinal electric field responsible for the observed proton acceleration.

In conventional studies of laser-driven proton acceleration, the longitudinal accelerating field is typically attributed to sheath fields generated by charge separation at the rear surface of the target, as described in the target normal sheath acceleration (TNSA) framework. In contrast, the present work proposes that transition radiation fields produced by relativistic electron bunches exiting the plasma can significantly contribute to, and even dominate, the accelerating field. This interpretation provides a new perspective on how relativistic electron dynamics may influence the acceleration environment, particularly in near-critical density targets where superponderomotive electrons are generated.

In the revised manuscript, the authors have provided substantially expanded analysis to support this interpretation. They introduce a theoretical framework that separates the

longitudinal electric field into Coulomb and transition-radiation components based on the dynamics of relativistic electron bunches. The comparison between this analytical treatment and 3D PIC simulation results strengthens the argument that the transition radiation field can play a dominant role under the conditions considered. In addition, the authors have also clarified several issues raised in the first review, including the THz-based estimation of the TRF amplitude and its uncertainties, the discussion of optimal foam thickness, and the assumptions used to distinguish TRF contributions from conventional sheath fields in the simulations. These revisions improve the overall clarity and balance of the manuscript.

Overall, my previous concerns have been satisfactorily addressed. I therefore believe the manuscript now meets the standards of Nature Communications and strongly recommend its publication.

Response: We sincerely thank the reviewer for the very positive and detailed evaluation of our revised manuscript. The reviewer has insightfully and comprehensively summarized the key innovations of our work. We are also grateful for the reviewer's constructive suggestions in the first round of review, which greatly improved the quality of our manuscript.

Reviewer #2

Comment 1: I appreciate that the authors have made a serious and constructive effort to address the concerns raised by both reviewers. In particular, it is clear that the ambiguity of the acceleration mechanism was one of the main weaknesses of the original manuscript, and the revised version responds to this issue in a much more substantial way. The added theoretical treatment and simulation analysis, especially the simplified scenario of a relativistic electron bunch traversing a plasma foil to separate Coulomb and radiative contributions, are compelling and significantly strengthen the paper.

These additions are valuable beyond the immediate manuscript, and the authors could consider retaining the key arguments in concise form in the main text while moving the more extended derivations, discussion, and supporting figures to a Supplementary Information file. This would help keep the manuscript focused and readable while still

making the full analysis accessible to interested readers.

The authors are also right that, in earlier studies of superponderomotive-electron–enhanced proton acceleration, the microscopic coupling between the electrons and the accelerating fields was often discussed only briefly. If the interpretation advanced here, that a significant part of what has previously been described as an "enhanced sheath" can in fact be understood more specifically in terms of transition-radiation fields generated by relativistic electrons, proves to be more generally valid, this would be an important observation for the community. In that sense, the manuscript has the potential to stimulate useful discussion and motivate more targeted experiments in the future.

Response: We are sincerely grateful for all the constructive suggestions you have provided over the two rounds of review, which have significantly strengthened the paper. We also thank you for your highly positive assessment of our work.

Following your suggestions, we have added additional content to the Supplementary Information. Specifically, we have included the theoretical framework for separating Coulomb and transition radiation fields based on electron dynamics; explanation of Equations (1-2); comprehensive 3D PIC simulations validating our model from non-relativistic to ultra-relativistic regimes and from idealized Gaussian bunches to realistic DLA-produced electron distributions; and a comparison with previous work concerning the "enhanced sheath" effect.

Comment 2: At the same time, some of the experimental correlations remain indirect, and ambiguities with respect to alternative or concurrent mechanisms persist. I therefore do not regard the present data as a definitive demonstration that TRF is the dominant mechanism in this specific case. That said, the level of evidence is broadly comparable to what has been accepted in other published studies of similar scenarios. Two points nevertheless deserve further attention. First, the statistical presentation of the experimental data should be reconsidered: standard deviations derived from only two or three shots are of limited significance, and it would be more informative to show the individual shots directly rather than only mean values with error bars. Second, it should be stated clearly in the main text, not only in the caption of Fig. 3, that the electron and terahertz signals were diagnosed independently in separate measurements and at different laser energies.

On balance, the revised manuscript now presents an interesting and physically well-motivated interpretation, supported by a much stronger theoretical and numerical framework, and I believe it merits publication and discussion by the community, provided the authors present their central claim with appropriate caution and do not overstate the exclusivity of the mechanism.

Response: We thank the reviewer for this constructive assessment.

According to your suggestion, we have replotted Figures 2(a) and 3(a) to present the results of protons and electrons of each shot, with the corresponding descriptions now included in the figure captions as well. Consistent trend is seen among the proton energy, the electron charge, the THz radiation versus the foam thickness.

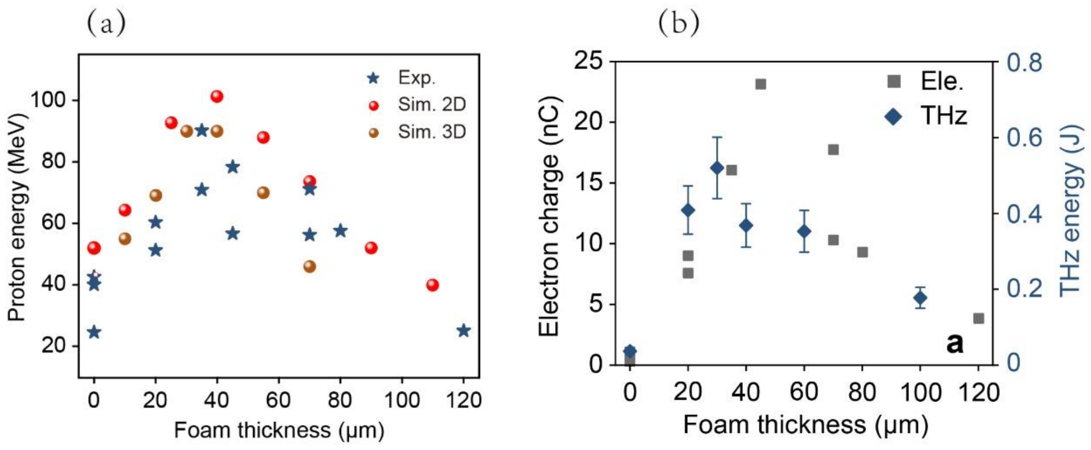

Fig. R1 (a) Proton cutoff energy (blue stars) and (b) electron charge (>16.2 MeV, gray dots) in individual shots.

Clear statement of separate measurements has been added in the main text. It should be clarified that, since only electron and THz data are shown in Fig. 3, the caption previously stated that these two were diagnosed independently. In fact, electrons and protons were measured simultaneously under identical conditions, while the THz measurement was performed at a laser energy of 68 J.

Changes:

Figs. 2(a) and 3(a), and the captions.

In the separate shots with higher laser energy of 68 J, we measured the TRF spectrum in frequency band of 0.1-15 THz as shown in Fig. 3.

The terahertz signals were diagnosed independently in separate shots with laser energy of

68 J

As you have previously noted and kindly acknowledged, the new understanding in this work regarding the energy coupling between superponderomotive electron bunches and the proton acceleration field represents a deeper insight into earlier related studies. In order to present our interpretation more cautiously and scientifically, we have further balanced the description of other mechanisms that may co-exist to address your concern about the exclusivity of the proposed mechanism, as seen in the following changes. This balanced description does not affect the core discussion of our manuscript.

Changes:

1) ~~When combined with the CSF,~~ This strong TRF jointly boosts the proton cut-off energy to 90 MeV, more than twice the energy obtained using TNSA alone.
2) Based on three-dimensional relativistic kinetic simulations, we decouple the TRF from the CSF, and that suggests TRF plays a ~~critical~~ non-negligible role in both enhancing the peak field strength and broadening the acceleration region.
3) Similar plateau spectra have been observed in magnetic vortex acceleration (MVA)[36, 37]. ~~However, this mechanism is not active in our setup.~~ Typical MVA requires the large-scale low-density plasma behind the target[36]. Here, the $500n_c$ substrate blocks the laser penetration and attenuates the magnetic field expanding from the plasma channel to the proton acceleration region. Moreover, MVA protons are highly collimated, with a typical full angle of 1–3° for high energy proton when using a high-power laser[38, 39]. In our experiment, the TPS was configured to diagnose only protons emitted at an angle of 5° with respect to the laser propagation direction. ~~The TPS in our experimental configuration makes it less likely to capture the highly directional and energetic protons.~~

Thank you again for your time. We hope that we have addressed all your concerns.
